# Dominant role of excitons in photosynthetic color-tuning and light-harvesting

**DOI:** 10.3389/fchem.2023.1231431

**Published:** 2023-10-16

**Authors:** Kõu Timpmann, Margus Rätsep, Arvi Freiberg

**Affiliations:** ^1^ Institute of Physics, University of Tartu, Tartu, Estonia; ^2^ Estonian Academy of Sciences, Tallinn, Estonia

**Keywords:** exciton transport, exciton bandwidth, purple photosynthetic bacteria, fluorescence excitation anisotropy, hole-burning

## Abstract

Photosynthesis is a vital process that converts sunlight into energy for the Earth’s ecosystems. Color adaptation is crucial for different photosynthetic organisms to thrive in their ecological niches. Although the presence of collective excitons in light-harvesting complexes is well known, the role of delocalized excited states in color tuning and excitation energy transfer remains unclear. This study evaluates the characteristics of photosynthetic excitons in sulfur and non-sulfur purple bacteria using advanced optical spectroscopic techniques at reduced temperatures. The exciton effects in these bacteriochlorophyll *a*-containing species are generally much stronger than in plant systems that rely on chlorophylls. Their exciton bandwidth varies based on multiple factors such as chromoprotein structure, surroundings of the pigments, carotenoid content, hydrogen bonding, and metal ion inclusion. The study nevertheless establishes a linear relationship between the exciton bandwidth and Q_y_ singlet exciton absorption peak, which in case of LH1 core complexes from different species covers almost 130 nm. These findings provide important insights into bacterial color tuning and light-harvesting, which can inspire sustainable energy strategies and devices.

## 1 Introduction

**FIGURE 1:**
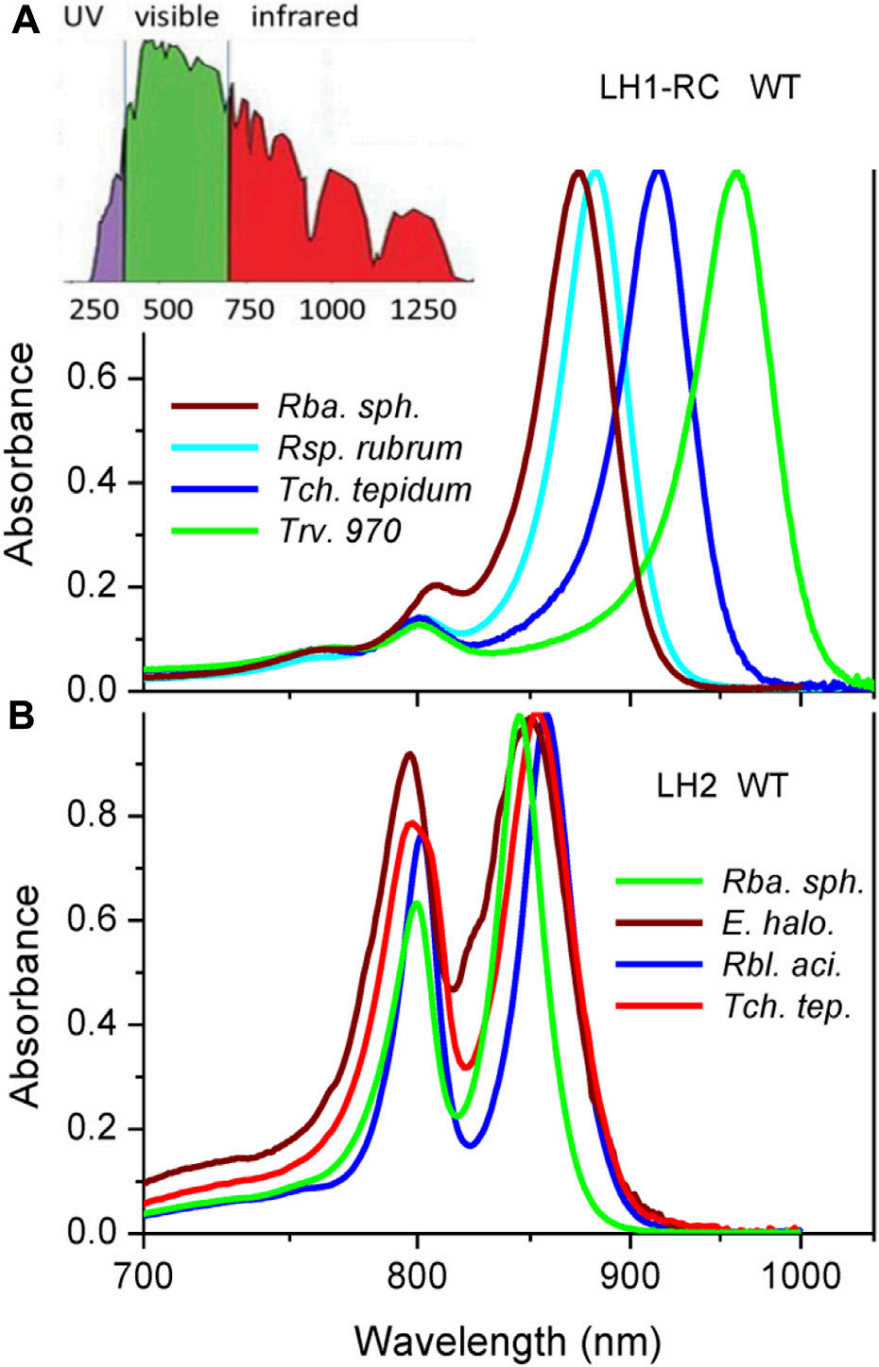
Peak-normalized Q_y_ absorption spectra of core LH1-RC **(A)** and peripheral LH2 **(B)** light-harvesting complexes from wild type purple bacteria recorded at ambient temperature of 295 K. For comparison sake, reciprocal (linear in energy) wavelength scale is applied. Shown in the inset of panel A is the reference terrestrial solar irradiation spectrum. The bacteria under study are evolved to efficiently exploit red-colored part of the solar spectrum.

Photosynthetic organisms—plants, algae, and some bacteria - use solar energy to convert the inorganic matter to organic compounds required for survival of all lifeforms on Earth (Blankenship, 2002). This basic task is carried out by a number of specialized protein units enriched by pigment chromophores, notably by chlorophylls in plants and algae, and bacteriochlorophylls in photosynthetic bacteria (Grimm et al., 2006). In the Earth’s crowded ecosystems, the ability for color adaptation is essential for common thriving of various photosynthetic species. This race is most evident in the red to near infrared part of the solar spectrum, where the absorption maxima of plants and algae at about 700 nm appear clearly separated from those of photosynthetic bacteria that span from about 800 nm to about 1,000 nm, see Figure 1.

Much of this almost 100-nm wide spectral gap is produced by different dominating light-harvesting chromophores, which in the former case is chlorophyll *a* (Chl) and in the latter case bacteriochlorophyll *a* (BChl). This explanation, however, is not fully satisfactory. For the first, the absorption band positions of native photosynthetic systems are significantly red-shifted (shifted towards longer wavelengths or lower energy) compared with the absorbance of Chl and BChl pigment molecules dissolved in normal solvents. At low concentration the main *Q*_y_ singlet absorption bands of Chl and BChl peak at 660 nm and 770 nm, respectively (Grimm et al., 2006; Taniguchi and Lindsey, 2021). Only long tails of these spectra reach the destined wavelengths (Leiger et al., 2020), but the weak absorbance there is not adequate for supporting effective photosynthesis.

The above systematic discrepancy known for ages has been motivating numerous studies to understand tuning mechanisms of the spectra of photosynthetic light-harvesting complexes. One mechanism universally observed in all native systems is aggregation of the pigments. Close proximity facilitates energy transfer. In photosynthetic chromoproteins, it also generally supports collective (delocalized) excited states of the pigments called excitons with stabilized energy, thus the red-shifted spectra (Sauer and Austin, 1978; Scherz and Parson, 1984; van Amerongen et al., 2000). The classical molecular exciton model developed for understanding the spectroscopy of simple molecular aggregates and molecular crystals (Davydov, 1971) involves separate terms of local site energies (transition energies of individual molecules) and inter-molecular (exciton) coupling energies. As a consequence, it is considered that the interactions between molecules and surroundings impact only site energies, leaving the exciton couplings unaffected, while the interactions between molecules modulate just exciton energies. However, straightforward application of this model to more complex photosynthetic pigment-protein complexes may not always be justified (Jang and Mennucci, 2018). Mixing of the neutral exciton states with charge-transfer states in closely coupled pigment associations constitutes modern variation of the same theme (Alden et al., 1997; Nottoli et al., 2018; Bourne-Worster et al., 2023).

Indeed, most of the mechanisms resulting in tuning the absorption spectra of photosynthetic chromoproteins such as hydrogen (H-) bonding (Fowler et al., 1994; Sturgis et al., 1997; Sturgis and Robert, 1997; Uyeda et al., 2010), axial ligation (Kania and Fiedor, 2006) or macrocycle ring distortions (Zucchelli et al., 2007; De Vico et al., 2018) simultaneously affect both site energies and exciton couplings. Structural factors that control the position of the Q_y_ absorption band of BChl in bacterial pigment-protein complexes have been reviewed, for instance, in (Cogdell et al., 2002). Besides, continuous fluctuations of the protein environment, thus also the conformations and transition dipole moments of the pigments, necessarily reflect on both site energies and inter-pigment couplings (May and Kühn, 2000; van Amerongen et al., 2000). Well-known consequences of this dynamics are shifts and broadenings of the exciton states/bands. Less recognized is that the coupling of light-harvesting excitons to fluctuating protein surroundings may lead to exciton self-trapping effect (Timpmann et al., 2001; Freiberg et al., 2003). Static disorder only facilitates this phenomenon (Trinkunas and Freiberg, 2006).

While the concept of photosynthetic excitons is widely recognized (van Amerongen et al., 2000), the implications of delocalized excited states on color tuning and excitation dynamics of light-harvesting complexes await to be better understood (Scholes et al., 2011; Fassioli et al., 2014; Mirkovic et al., 2017; Sohail et al., 2017; Reimers et al., 2020; Cignoni et al., 2022). One of the main obstacles in achieving this fundamental photobiological goal is the lack of solid experimental data on coupling energies of photosynthetic excitons. To get this information, one as a minimum requires to determine spectral positions of the states that correspond to the high-energy top and low-energy bottom of the manifold of exciton states simply termed as exciton band. This seems easy, but due to structural and other constraints (such as overlapping spectra of different chromoprotein complexes, see below), the top and bottom states are simultaneously rarely available by conventional absorption measurements, requiring application of complementary methods. The methods previously utilized for the study of exciton band structure in separate purple bacterial complexes comprise hole-burning (Reddy and Small, 1991), circular dichroism (CD) (Koolhaas et al., 1998; Georgakopoulou et al., 2006b), non-linear absorption (Leupold et al., 1999), fluorescence excitation anisotropy (Timpmann et al., 2004; Timpmann et al., 2005), two-photon excitation (Stepanenko et al., 2012; Razjivin et al., 2019), transient absorption kinetics (Gall et al., 2010), two-dimensional electronic spectroscopy (Dahlberg et al., 2016), and self-modeling spectrum fitting (Trinkunas et al., 2012).

A centerpiece of the bacterial photosynthetic machinery is the LH1-RC super-complex, where the light-harvesting 1 (LH1) complex directly encircles the reaction center (RC) complex (Hunter et al., 2008). In order to cope with the great variability of terrestrial solar irradiation intensity, many purple bacteria additionally develop peripheral or LH2 light-harvesting complexes. The LH2 complex, usually not in straight contact with the RC, transfers its energy to the RC via the LH1 complex (Freiberg et al., 1989; van Amerongen et al., 2000). Both LH1 and LH2 appear as oligomers of basic heterodimeric structures composed of membrane-spanning α-helical α- and β-polypeptides, with each apoprotein unit noncovalently binding three (LH2) or two (LH1) BChl molecules and one (LH2) or two (LH1) carotenoid molecules. The number of apoprotein units and large-scale architecture of the complexes is species dependent, see (Gardiner et al., 2020; Kimura et al., 2023) for recent reviews. In the wild type (WT) *Rhodobacter (Rba.) sphaeroides*, for example, the presence of additional pufX polypeptide cuts the apoprotein circle and leads most of the core complexes to assemble into a nonplanar S-shaped array of 28 αβ-BChl_2_ structural units that enclose 2 RCs (Qian et al., 2021a; Cao et al., 2022). This is the so-called dimeric core complex. In photosynthetically grown cells the dimeric antenna structures coexist with open-ring monomeric structures comprising 14 αβ-BChl_2_ subunits (Qian et al., 2021b). The absence of pufX in certain native and mutant complexes yields 16 αβ-BChl_2_ structural elements of LH1 fully encircling a single RC, the planar monomeric core complex LH1-RC (Jones et al., 1992). In LH2, a closed planar ring of apoproteins in the membrane plane is rather typical, albeit the complexes may involve different numbers of subunits counting from 7 (Gardiner et al., 2021) to 13 (Kereïche et al., 2008). The lone BChl pigment cofactor close to cytoplasmic surface of LH2 and a pair of BChls nearer to periplasmic surface form two circular pigment arrangements, named B800 and B850 according to their respective Q_y_ exciton absorption band positions at ambient temperature. Carotenoid molecules structurally interconnect the B850 and B800 pigment assemblies. Basic quantum mechanical models to understand spectroscopy of cyclic light-harvesting complexes of purple bacteria can be found in (Novoderezhkin and Razjivin, 1995; van Amerongen et al., 2000; Hu et al., 2002; Cogdell et al., 2006).

In the current work, we first aimed at evaluation of the top and bottom edges, thus the bandwidth of LH1 and LH2 light-harvesting excitons, for the broad class of photosynthetic purple bacteria comprising purple sulfur and purple non-sulfur bacteria. We show that this task is feasible by simultaneous utilization of a variety of spectroscopic techniques, including absorption, fluorescence excitation, fluorescence excitation anisotropy, circular dichroism (CD), and spectral hole-burning. The bandwidths of excitons were determined for 17 core and 16 peripheral WT and engineered light-harvesting detergent-purified and/or membrane-embedded complexes. A robust linear correlation established between the bandwidths and positions of the Q_y_ exciton absorption spectra reveals key role of excitons in enhancing the spectral range of bacterial light-harvesting, confirming the earlier cherished intuition (van Amerongen et al., 2000).

## 2 Materials and methods

### 2.1 Materials

The various LH1 and/or LH1-RC core, and peripheral LH2/LH3 complexes from sulfur (*Ectothiorhodospira (E.) haloalkaliphila, Thermochromatium (Tch.) tepidum*, *Thiorhodovibrio* strain 970 *(Trv.* 970*)*) and non-sulfur (*Rba. sphaeroides, Rhodoblastus (Rbl.) acidophilus, Rhodospirillum (Rsp.) rubrum*) purple bacteria studied here were kindly donated to the authors by Profs. R. Cogdell (Glasgow University), C. N. Hunter (Sheffield University), A. A. Moskalenko (IBBP, Pushchino), Z.-Y. Wang-Otomo (Ibaraki University), and N. Woodbury (Arizona State University). The purple sulfur and purple non-sulfur bacteria, respectively, use either sulfide and hydrogen or organic compounds as an electron donor (Hunter et al., 2008). Isolation and purification of the complexes were carried out following standard protocols, as described in refs. provided in bookkeeping Table 1. Specific samples investigated are enlisted in Table 2 and Table 3. Distinct aspects of the sample preparation and handling are commented at proper places of the manuscript.

**TABLE 1:** List of the BChl-a containing light-harvesting complexes studied in this work.

Strain		Complex	
		LH1-RC	LH2
*Trv*. 970^c1^	Sulfur bacteria	×	N/A^a^
*Tch. tepidum* ^c2^		×	×
*E. haloalkaliphila* ^c3^		NA^b^	×
*Rsp. rubrum* ^c4^	Non-sulfur bacteria	×	N/A^a^
*Rba. sphaeroides* ^c5,c6,c7^		×	×
*Rbl. acidophilus* ^c8^		×	×

^a^ N/A–not applicable.

^b^ NA-not available.

^c^ References: 1- (Tani et al., 2020), 2- (Suzuki et al., 2007), 3- (Ashikhmin et al., 2014), 4- (Moskalenko et al., 1996), 5- (Sturgis et al., 1997), 6- (Jones et al., 1992), 7- (Olsen et al., 1994), 8- (Cogdell and Hawthornthwaite, 1993).

**TABLE 2:** The Q_y_ exciton band parameters for detergent-isolated and membrane-embedded (m-) core light-harvesting complexes recorded at 4.5 K.

Strain	Sample	Absorption peak (nm)^a^		Anisotropy dips (nm)		Exciton bandwidth (cm^–1^)^b^		Γ_IDF_ (cm^–1^)^c^
		λ_Q_	λ_0_	Blue	Red	∆E	∆E_0_	
*Trv.* 970	LH1-RC WT^d1^	986.8	1,002.8	770.6	977.9	2,751	3,005	88
	LH1-RC (Ca−)^d1^	900.6	928.5	757.9	894.8	2,020	2,424	218
*Tch. tepidum*	LH1-RC WT^d1^	938.4	951.3	765.9	930.8	2,310	2,545	117
	LH1-RC (Ba+)	905.9	924.1	760.5	900.1	2,039	2,328	198
*Rsp. rubrum*	m-LH1-RC WT	897.4	909.8	755.0	889.1	1,998	2,254	123
	m-LH1-RC G9+^e^	888.6	903.1	754.9	883.4	1,927	2,174	155
*Rbl. acid*	m-LH1-RC WT^d2,d3^	901.8	NA	765.6	901.6	1,969	NA	NA
*Rba. sphaeroides*	LH1^d3,d4^	886.1	896.0	756.0	880.0	1,864	2,067	119
	m-LH1^d3,d4^	888.4	900.1	749.7	883.8	2,024	2,229	108
	LH1-RC-pufX WT^d3,d4,^ ^f^	884.0	894.6	749.8	873.3	1,886	2,159	128
	m-LH1-RC-pufX WT^d3,d4,^ ^f^	885.6	897.6	751.5	876.1	1,892	2,166	118
	m-LH1-RC-pufX WT^g^	886.1	897.8	755.1	876.4	1,833	2,105	135
	m-LH1-RC-pufX ΔCrtB^e^	883.0	897.5	755.1	877.7	1,850	2,101	109
	LH1-RC-pufX^h^	861.1	872.8	751.0	855.5	1,627	1,857	186
	LH1-RC-pufX^i^	880.3	892.4	744.4	867.2	1,902	2,228	160
	LH1-RC^d4,^ ^j^	884.5	897.6	752.9	879.6	1,913	2,141	134
	m-LH1-RC^d4,^ ^j^	885.6	898.0	751.6	880.8	1,952	2,169	120

^a^ λ_Q_ and λ_0_, respectively, represent the peak wavelength of the Q_y_ absorption band and the maximum of the inhomogeneous distribution function (IDF) of the k = 0 exciton states; experimental uncertainty of the measurements: ±(0.4–0.8) nm.

^b^ Uncertainty of the ∆E and ∆E_0_ values: ±20 cm^−1^.

^c^ Γ_IDF_, full width at half maximum of IDF; uncertainty of the measurements: ±(10–30) cm^−1^.

^d^ References: 1- (Timpmann et al., 2021), 2- (Kunz et al., 2013), 3- (Freiberg et al., 2013), 4 - (Timpmann et al., 2005). For comparability sake, the data obtained in previous publications have been re-scaled according to the experimental and calculation procedures taken in the present work.

^e^ Carotenoid-less mutant.

^f^ Dominant carotenoid spheroidenone.

^g^ Dominant carotenoid spheroidene.

^h^ α+11 H-bond mutant.

^i^ β+9 H-bond mutant.

^j^ Monomeric closed-ring mutant.

**TABLE 3:** Exciton parameters related to the B850/B820 band of detergent-isolated and membrane-embedded peripheral light-harvesting complexes recorded at 4.5 K.

Strain	Sample	Absorption peak (nm)^a^		Anisotropy dips (nm)		Exciton bandwidth (cm^–1^)^b^		Γ_IDF_ (cm^–1^)^c^
		λ_Q_	λ_0_	Blue	Red	∆E	∆E_0_	
*Tch. tepidum*	LH2 WT	875.5	898.4	760.8	865.5	1,590	2,013	178
	LH2 (Ca removed)	NA	NA	760.5	865.5	1,597	NA	NA
	LH2 (Ba exchanged)	NA	NA	761.6	866.7	1,592	NA	NA
*E. haloalkaliphila*	LH2 WT	862.1	878.8	759.1	850.7	1,418	1,794	254
	LH2 (car-less)	860.5	886.8	757.4	851.6	1,460	1,926	240
*Rbl. acidophilus*	LH2 WT^d1,d2,d3,d4^	870.0	885.0	766.5	861.7	1,442	1,747	120
	m-LH2 WT^d1,d4^	870.4	NA	766.7	863.9	1,471	NA	NA
	LH2 WT (in PVA)^d4^	855.6	NA	761.4	847.5	1,332	NA	NA
	LH3 (B800-820)^d2^	821.4	836.0	738.2	816.5	1,300	1,584	240
*Rba. sphaeroides*	LH2 WT^d1,d5,^ ^e^	851.0	864.9	763.2	844.1	1,254	1,542	148
	m-LH2 WT^d1,^ ^e^	853.1	870.0	763.2	845.8	1,279	1,608	202
	LH2^f^	849.7	866.4	762.0	844.5	1,282	1,581	150
	LH2 (B800-less)^d5,^ ^f^	856.8	873.6	758.1	851.8	1,451	1,744	151
	m-LH2 (B800-less)^f^	859.4	877.5	759.5	853.6	1,452	1,771	241
	m-LH2^g^	838.3	855.8	758.3	836.6	1,234	1,502	210
	m-LH2^h^	827.2	845.7	759.2	826.2	1,068	1,347	232

^a^ Experimental uncertainty of the measurements: ±(0.5–0.8) nm.

^b^ Uncertainty of the ∆E and ∆E_0_ values: ±20 cm^−1^.

^c^ Experimental uncertainty of the measurements: ±(10–38) cm^−1^.

^d^ References: 1- (Freiberg et al., 2013), 2- (Freiberg et al., 2010), 3- (Pajusalu et al., 2011), 4- (Kunz et al., 2013), 5- (Timpmann et al., 2004). For comparability sake, the data obtained in earlier publications have been re-scaled according to the experimental and calculation procedures taken in the present work.

^e^ Dominant carotenoid spheroidenone.

^f^ Neurosporene mutant.

^g^ α–H-bond mutant.

^h^ αβ–H-bond mutant.

The concentrated samples were stored at −78°C in deep freezer. Prior the use the samples were diluted with buffer (20 mM Tris-HCl or 20 mM HEPES, depending on samples) to meet the required for specific measurements optical density. The buffers for purified complexes additionally contained proper detergent (n-dodecyl β-D-maltopyranoside (DDM), dihexanoylphosphatidylcholine (DHPC), octyl β-glucoside (βOG) or lauryldimethylamine oxide (LDAO)) to prevent aggregation of the proteins. The *Trv*. 970 and *Tch. tepidum* solutions also contained 60 mM CaCl_2_ to keep samples saturated with Ca^2+^. To produce the Ca^2+^-depleted samples, EDTA was added and the sample solution was incubated for several hours before the measurements. Glycerol with a 2:1 volume ratio was used to obtain transparent glassy samples at cryogenic temperatures.

### 2.2 Spectroscopy

xThe multipurpose spectroscopic setup applied in this work has been recently described (Rätsep et al., 2017; Timpmann et al., 2021). It comprises a model 3900S Ti: sapphire laser of 0.5 cm^–1^ linewidth pumped by a Millennia Prime solid-state laser (both Spectra Physics), a 0.3-m focal length spectrograph Shamrock SR-303i, equipped with a thermo-electrically cooled CCD camera DV420A-OE (both Andor Technology) for the measurements of fluorescence excitation anisotropy and hole-burning spectra, and a high-stability broad-band tungsten light source BPS100 (BWTek) for absorption measurements. In the measurements of anisotropy spectra, the vertically polarized laser beam was scanned over a proper wavelength range. The fluorescence spectra were detected through high-contrast analyzing polarizer set parallel or perpendicular to the excitation laser beam. Another correcting polarizer was fixed at 45° to the polarization direction of the analyzing polarizer. The estimated experimental uncertainty of the baseline anisotropy value is ± 0.03. Photobleaching and scattering are potentially detrimental to the quality of anisotropy measurements. To minimize photobleaching, the excitation light flux density was kept as low as feasible for achieving reasonable signal-to-noise ratio. At the experimental power density of 1–10 mW/cm^2^ some amount of photobleaching was only observed when excited in resonance with the main absorption band of the samples, which, however, didn’t concern determination of the anisotropy dip positions. Optimization of the sample concentration and blackening of the cuvettes’ holder were used to curtail the excitation laser-light scattering. The wavelength scale of the spectrometer was determined with a precision of ± 0.1 nm using a Ne−Ar calibration lamp. In low-temperature measurements the PMMA plastic cuvettes (Brand) filled with the sample solution were placed into a liquid helium bath cryostat (Utreks). The cryostat was equipped with a temperature controller (model 211 Lakeshore Cryotronics), stabilizing the temperature within ± 0.5 K.

### 2.3 Data analysis

The data were analyzed and fitted using the graphing and data analysis software Origin 9.0 SR1 (OriginLab).

## 3 Results and discussion

For an overview and reading convenience, we will start this Section by presenting the absorption and fluorescence excitation anisotropy spectra of the core (LH1 or LH1-RC) and peripheral (LH2) light-harvesting complexes from WT purple bacteria. We then continue with describing the modifications of these basic spectra caused by the changes of the chromoprotein structure such as due to H-bonding engineering or of environment resulting a detergent purification process. Other spectra/methods will be introduced in proper places. Most of the spectra presented are recorded at low temperature of 4.5 K for the sake of improved spectral resolution. Numerical data characterizing excitons in LH1/LH1-RC and LH2 are, respectively, collected into Table 2 and Table 3.

### 3.1 Absorption spectra of wild type complexes

Demonstrated in Figure 1 are the Q_y_ absorption spectra of LH1-RC (Figure 1A) and LH2 (Figure 1B) complexes from selected WT bacteria in the proper light-harvesting exciton spectral range, see below. Broadband version of the absorption spectra spanning from 250 nm to 1,100 nm can be found in, Supplementary Figure 1. For better comparison, reciprocal (linear in energy) wavelength scale is applied whenever feasible. Also, for the convenience sake, a reference terrestrial solar irradiation spectrum is provided on top of the experimental absorption spectra. It is well-known that the absorption spectra of LH1-RC complexes are in the near-infrared part of the solar spectrum dominated by a sole Q_y_ exciton band of LH1, also known as B875. The RC cofactors contribute visibly into this spectrum only around 750 and 850 nm, in the high-energy tail of the absorbance of LH1 excitons. Absorption spectra of LH2 complexes encompass two peaks, due to weakly-coupled and strongly-coupled pigments in the B800 and B850 circular arrangements of BChls, respectively.

The peak-normalized spectra in Figure 1 demonstrate an exciting spectral tunability of LH1 excitons in different native complexes that almost reaches 100 nm. This is much more than the extent observed in the case of B850 (12 nm) or B800 (5 nm). For the sake of comparison, it is interesting to note that the variations of the Q_y_ energy of BChl in polar and nonpolar solvents are about 17 nm and 22 nm, respectively (Limantara et al., 1997). As follows, we will concentrate on the properties of the most responsive strongly coupled B875 and B850 excitons.

### 3.2 Fluorescence excitation anisotropy spectra of wild type complexes

**FIGURE 2:**
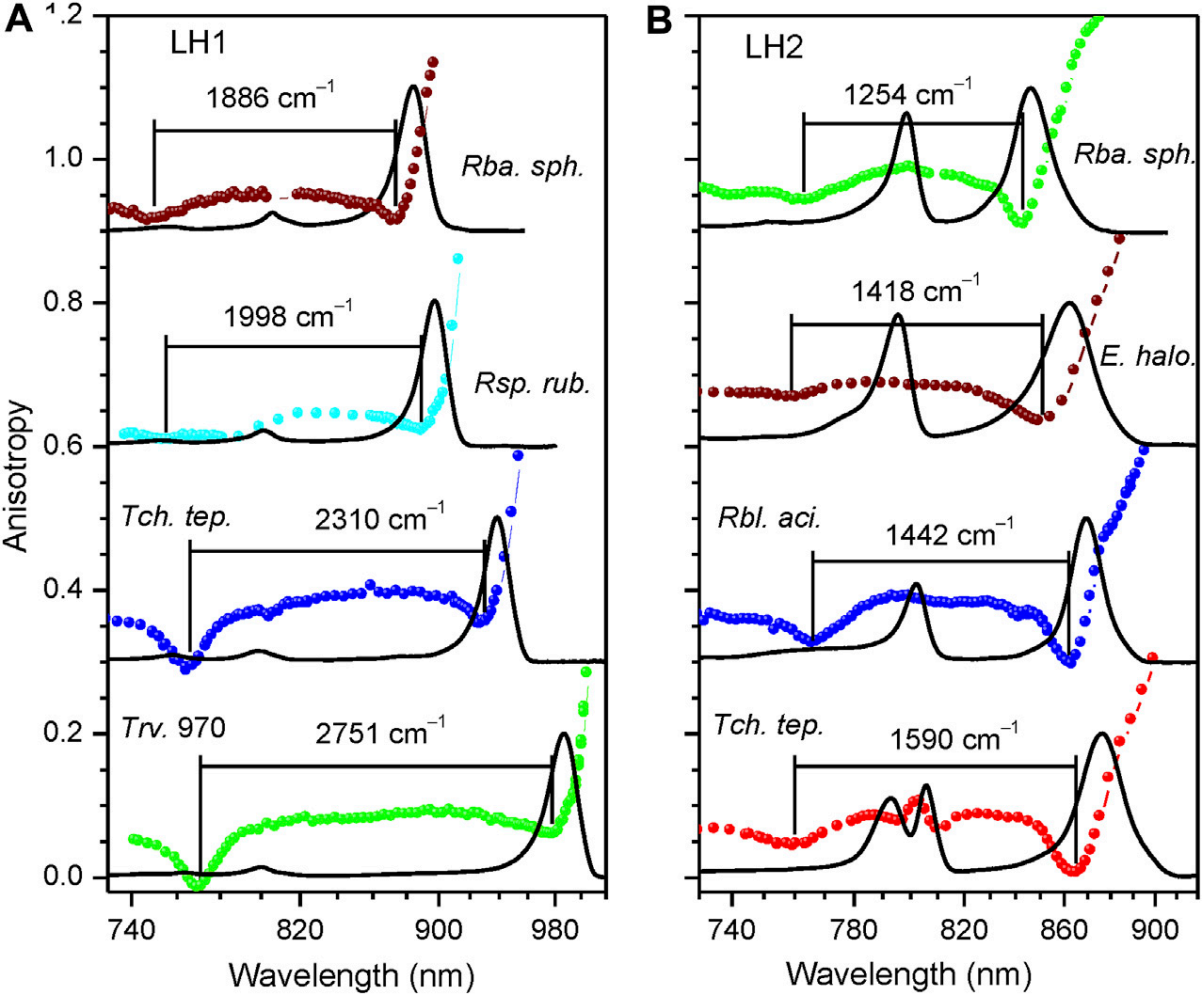
Fluorescence excitation anisotropy (colored data points, same color coding as in Figure 1) and absorption (black line) spectra recorded at 4.5 K of *Q*_y_ excitons in LH1/LH1-RC **(A)** and LH2 **(B)** complexes of wild type bacterial strains as indicated. Shown in the background are absorption spectra of the samples. The spectra corresponding to different complexes are vertically shifted relative to each other by 0.3 anisotropy units for better visibility. Lines connecting discreet anisotropy data points are here and subsequently drawn for leading the eye. The numbers on top of anisotropy curves indicate ∆E exciton bandwidths.

Presented in Figure 2 are the fluorescence excitation anisotropy spectra of the Q_y_ excitons in, respectively, LH1/LH1-RC (Figure 2A) and LH2 (Figure 2B) complexes from the same WT bacterial strains as exposed in Figure 1. Shown also with continuous line is absorption spectra of the samples.

**FIGURE 3:**
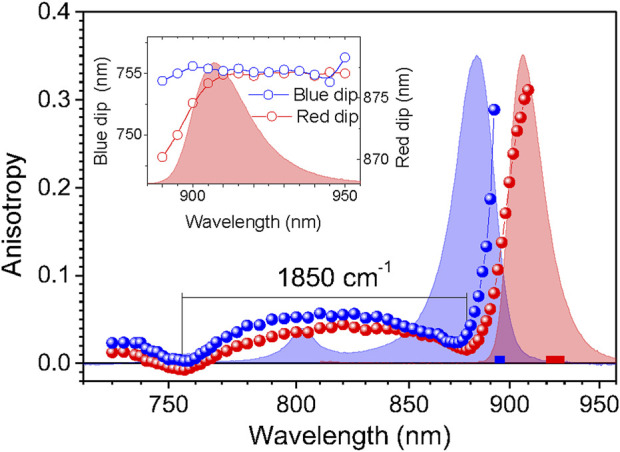
Variations of the fluorescence excitation anisotropy spectra in dependence of the fluorescence recording wavelength represented in case of the m-ΔCrtB carotenoid-less LH1-RC mutant membrane complex of *Rba. sphaeroides*. The anisotropy data designated by blue and red balls are collected selectively, using parts of the emission spectrum indicated by correspondingly colored bars. Exposed in the background are the absorption (blue line) and fluorescence (red line) spectra of the sample. Shown in the inset is the dependence of the blue (left axis) and red (right axis) anisotropy dip positions on the fluorescence recording wavelength.

The shape of the anisotropy spectrum generally depends on the recording wavelength, see Figure 3. Being an evidence of a spectrally disordered ensemble of light-harvesting complexes, this effect is the strongest at selective recording in the blue side of the emission spectrum, but almost vanishes when recording at red part, past the maximum of the spectrum, as demonstrated in the inset of Figure 3. Therefore, in order to handle comparable results, selective recording of fluorescence at red side of the spectra with 10-nm bandwidth was commonly applied in the anisotropy measurements.

In agreement with the previous work (Timpmann et al., 2004; Timpmann et al., 2005), the anisotropy spectra of different cyclic light-harvesting complexes look rather similar. They feature high anisotropy values at the long wavelength edge of the respective absorption spectra that strive towards a theoretical maximum value of 0.4 and two dips towards shorter wavelengths, where anisotropy values close to 0 are generally observed. According to numerical modeling (Trinkunas and Freiberg, 2006; Pajusalu et al., 2011) the higher-energy (or blue) anisotropy dip consistently showing up in a relatively narrow spectral range of 760 ± 10 nm rather precisely reproduces the high-energy edge of the Q_y_ exciton band, whereas the red dip appears around the strong (k = ±1, ±2) Q_y_ exciton transitions.

Hence, the energy difference ∆E between the blue and red dips, although underestimating the actual exciton bandwidth, can be considered as an effective measure of the exciton bandwidth in cyclic light-harvesting complexes (Timpmann et al., 2004). As shown in (Timpmann et al., 2005), a more precise estimation of the exciton bandwidth is obtained as the energy difference ∆E_0_ between the blue dip of the anisotropy spectrum and λ_0_. The λ_0_ represents the peak wavelength of the maximum of the inhomogeneous distribution function (IDF) of the lowest-energy k = 0 exciton states, which is measured as the peak position of the hole burning action spectrum (Reddy and Small, 1991). From the ∆E and ∆E_0_ data collected in Table 2 and Table 3, one can immediately grasp that exciton bandwidths in peripheral complexes are systematically narrower compared with those in core complexes, the ratio changing from about 2/3 in *Rba. sphaeroides* to almost 3/4 in *Rbl. acidophilus*.

### 3.3 Impact of the chromoprotein environment and structure

**FIGURE 4:**
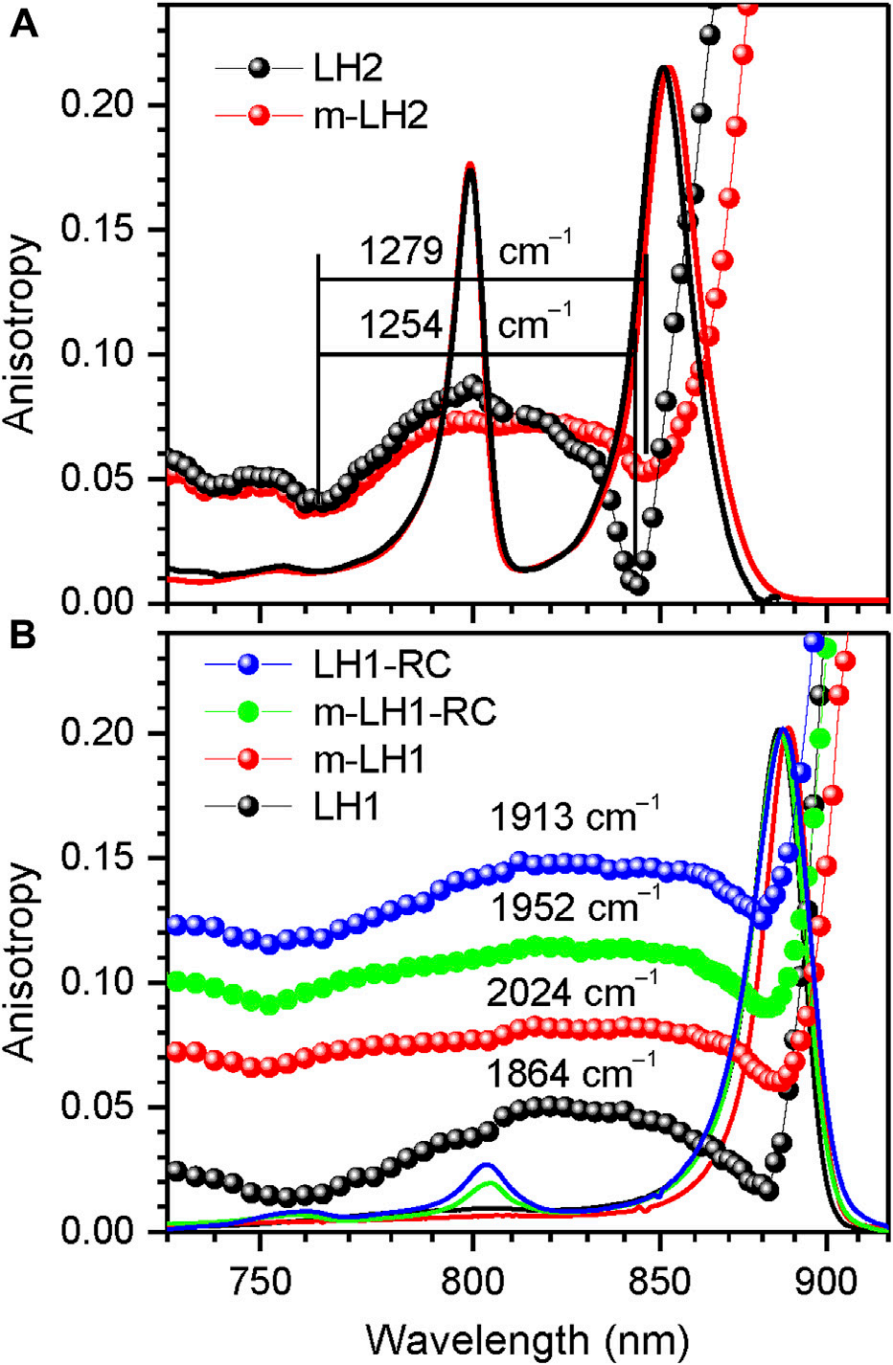
Comparison of the fluorescence excitation anisotropy spectra (scattered data points) of Q_y_ excitons in detergent-isolated and membrane-embedded LH2 **(A)** and LH1/LH1-RC complexes **(B)** of *Rba. sphaeroides*, as indicated. The multiple anisotropy spectra in panel **(B)** are for better visibility up-shifted with respect to the bottom LH1 spectrum by 0.06 anisotropy units in case of m-LH1, by 0.085 units in case of m-LH1-RC, and by 0.10 units in case of LH1-RC. Shown also with continuous lines are the correspondingly colored and peak-normalized absorption spectra of the samples.

Generally speaking, molecular excitons are sensitive to all physical factors that disturb transition energies of the sites and couplings between them. As follows, we will demonstrate using parallel measurements of fluorescence anisotropy and absorption spectra the responses of the light-harvesting exciton band structure upon altering the environment and internal structure of the chromoproteins. The problem is that in intricate systems such as the chromoproteins studied here, these effects can but rarely be uniquely unraveled. The answer to the question whether one is dealing with environmental or structural property depends on the degree of the effects observed as well as on subjective viewpoint of the investigators. Nevertheless, most researches agree that purification of membrane proteins out of its native membrane by mild detergents, a standard biochemical procedure for studying membrane proteins in isolation, provides a more-or-less pure case of an environmental change, see Figure 4.

**FIGURE 5:**
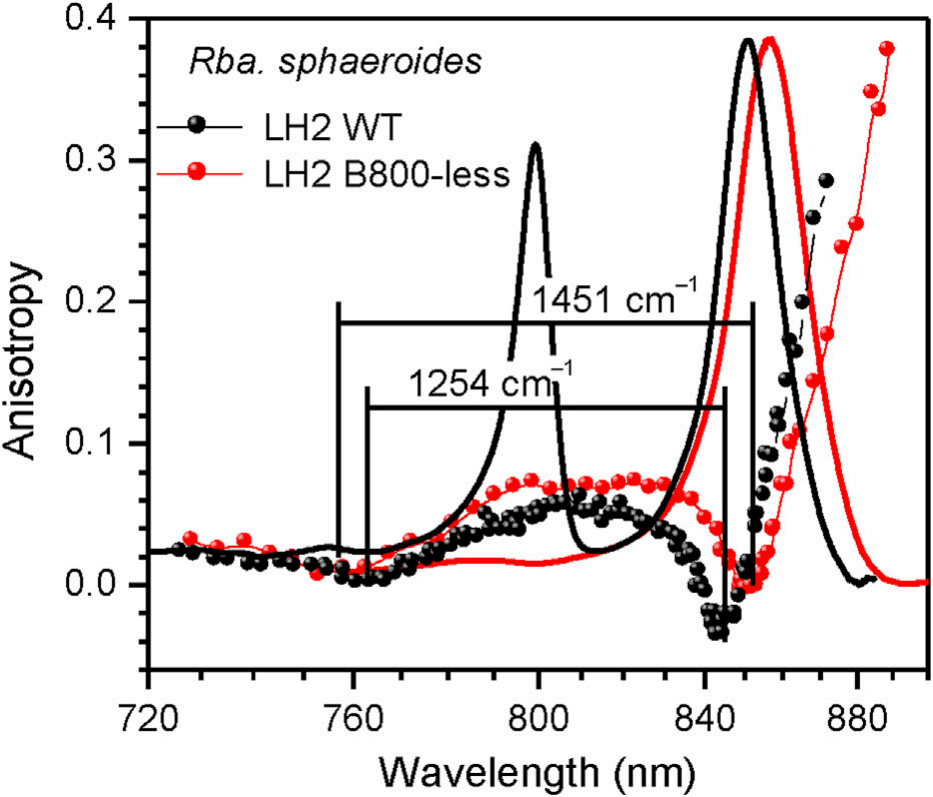
Comparison of the fluorescence excitation anisotropy spectra (scattered data points) of Q_y_ excitons in wild type (black) and B800-less (red) mutant LH2 complexes from *Rba. sphaeroides*. Shown in the background are correspondingly colored absorption spectra of the samples.

**FIGURE 6:**
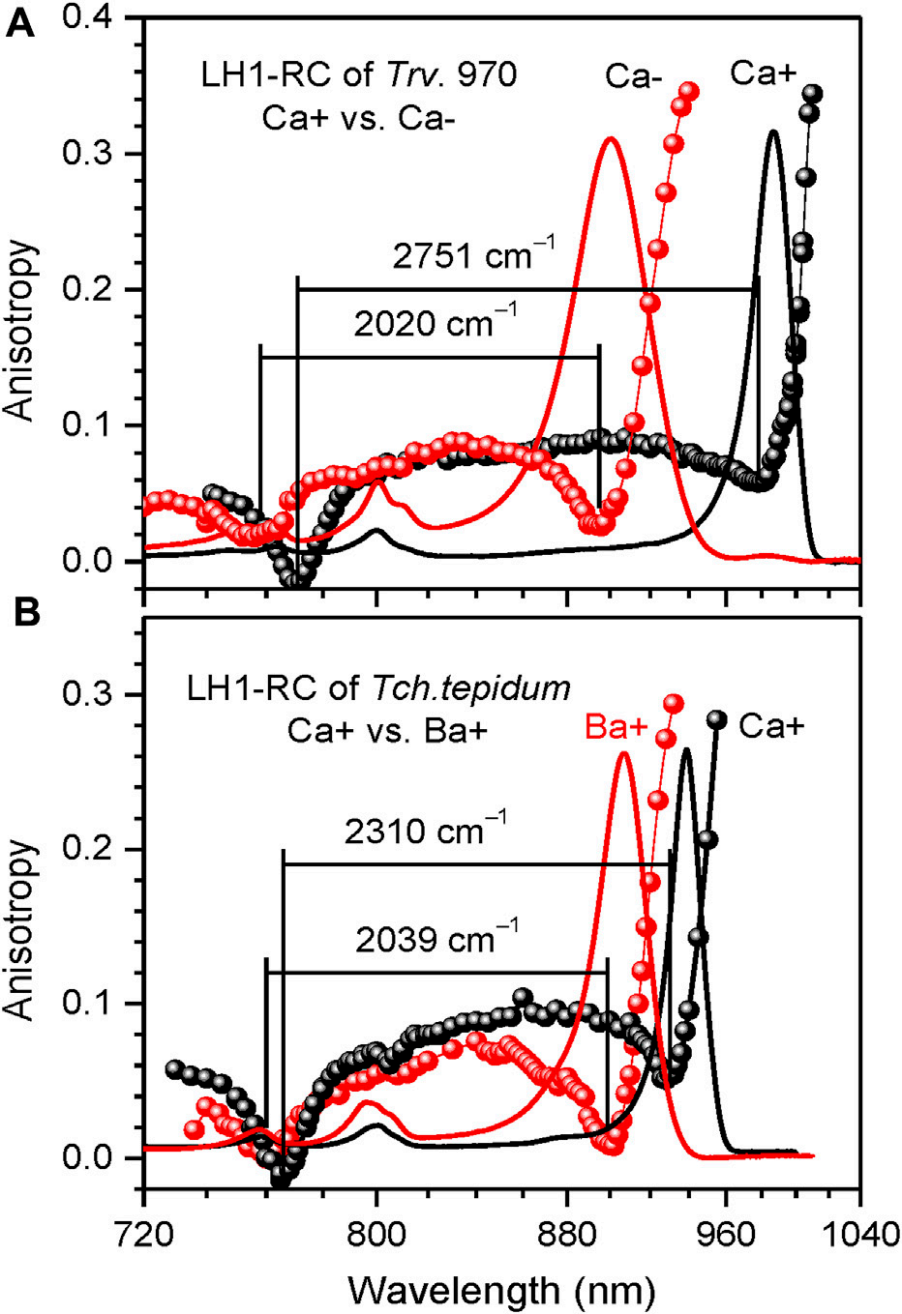
Comparison of the fluorescence excitation anisotropy spectra (scattered data points) of *Q*_y_ excitons in WT Ca-containing (black) and Ca-depleted (Ca–, red) LH1-RC complexes of *Trv.* 970 **(A)**, and in the complexes of *Tch. tepidum* containing either Ca^2+^ (Ca+, black) or Ba^2+^ ions (Ba+, red) **(B)**. Shown in the background are correspondingly colored absorption spectra of the samples.

**FIGURE 7:**
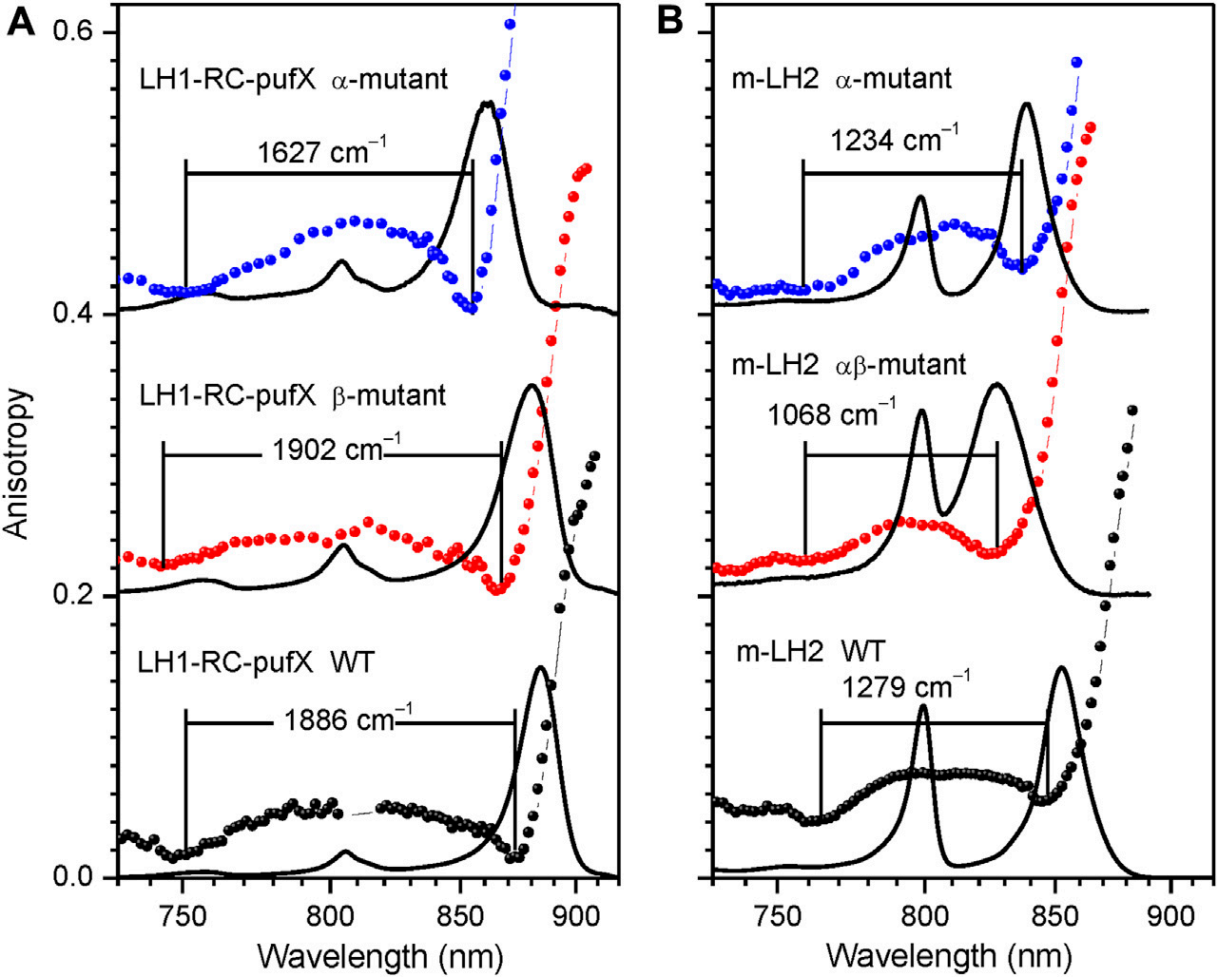
Assessment of the fluorescence excitation anisotropy spectra (scattered data points) of the *Q*_y_ excitons in native (black) and specific H-bond mutant LH1-RC-pufX **(A)** and LH2 **(B)** complexes (blue and red) from *Rba. sphaeroides* as indicated. Drawn by black line in the background are absorption spectra of the samples, all recorded at 4.5 K. See text for further explanations.

Examples of the internal structure variations presented in this work include changes in spatial organization of pigment chromophores illustrated by Figure 4B and Figure 5, in the content of metal ions (Figure 6), in the tertiary structure H-bonding network (Figure 7), and in the type and presence of carotenoids (Supplementary Figure 2).

**Native membrane-embedded *versus* detergent-isolated chromoproteins**. Shown in Figure 4A are effects of detergent-purification on the absorption and fluorescence anisotropy spectra of LH2 complexes of *Rba. sphaeroides*. As seen, the purification result in subtle but characteristic spectral modifications, which foremost include a couple of nanometers blue-shift and broadening of the Q_y_ absorption band. The Q_y_ band broadening observed may be caused by 1) the less ordered detergent environment or by 2) the enhanced exciton coupling. The concurrently measured anisotropy spectra that demonstrate narrowing of the exciton band exclude option 2). The weakening of exciton couplings upon the membrane protein purification is also consistent with the common-sense loosening of the protein structure in the surroundings of detergent micelle rather than with tightening. At the same time, as seen in Table 3, choice 1) appears to contradict with the much narrower IDF observed in the case of detergent-isolated sample. An obvious solution to this conundrum is that Γ_IDF_ in native membrane-embedded LH2s is dominated by external disorder, while that in detergent isolated LH2s, by internal disorder. See continuation of this discussion in Section 3.5.

The Q_y_ band of LH1 complexes similarly broadens in response to detergent-purification, while the exciton band is narrowing (Figure 4B). Yet in this case, internal disorder appears to play leading role because of greater Γ_IDF_ recorded for purified LH1 complexes, see Table 2. Notable also is the much-shifted position of the blue anisotropy dip and somewhat distorted structure of the anisotropy spectrum of purified LH1 complexes compared with those of membrane complexes. Especially the deviating blue anisotropy dip, which we will repeatedly meet below (see Figures 5–7), is a sure indicator of structural rearrangements accompanying the various sample handling processes (Freiberg et al., 2010; Kunz et al., 2013). Spectral responses following the assembly of LH1 with RC to mimic core LH1-RC complexes in the native membrane environment appear rather small and uncharacteristic, as are the differences between the spectra of native dimeric (LH1-RC-pufX) and monomeric mutant (LH1-RC) core complexes, see Figure 4B and Table 2.

Although subtle, important effects of embodiment of bacterial light-harvesting complexes into lipid bilayer have been thus established combining absorption, fluorescence anisotropy and hole-burning techniques. These data add to the mounting previous evidence that some photosynthetic processes may proceed differently in the native membrane, as compared to artificial detergent environment (Bowyer et al., 1985; Hunter et al., 1985; Beekman et al., 1997; Pugh et al., 1998; Urboniene et al., 2007; Pflock et al., 2008; Freiberg et al., 2012b; Freiberg et al., 2016). According to data in Table 2 and Table 3, the relative exciton band narrowing observed in LH2 and LH1 complexes of different organisms is rather small: 2%–3% and 3%–9%, respectively. In water-soluble chlorophyll proteins, for example, much stronger environmental effects have been detected (Fresch et al., 2020).

**LH2 complex with depleted B800 pigment ring**. Figure 5 offers a comparison of the fluorescence excitation anisotropy spectra of Q_y_ excitons for the WT and mutant B800-less complexes of LH2 from *Rba. sphaeroides*. Removal of the BChls from the B800 binding sites implicates large perturbation on both the protein spatial structure and the B850 exciton structure, as compared the same characteristics in WT complex. In conformity with this, a 5–6 nm red-shift of the B850 absorption band was observed, associated with almost 200 cm^–1^ broadening of ∆E (see Table 3).

Unfortunately, because of high affinity of B800 binding sites to native as well as to non-native pigments (Swainsbury et al., 2019), the ambition to prepare the complexes completely free from the B800 site inclusions is untenable. As an example, all the multiple samples in our disposal show different degree of contamination of the B800 sites with the BChl pigments, as shown in Supplementary Figure 3. To our mind, this provides a reasonable explanation for the long-lasting confusion about the variability of the relevant literature data (Leupold et al., 1999; Timpmann et al., 2004; Gall et al., 2010). In particularly, this removes any credibility behind the identification of an upper exciton component in the LH2 complex with depleted B800 of *Rba. sphaeroides* using CD (Koolhaas et al., 1998).

**Effects of structurally relevant metal ion inclusions**. Metal ions are frequent embellishments of photosynthetic proteins. However, to the best of our knowledge, only sulfur purple bacteria utilize Ca^2+^ ions to gain supremacy by extending LH1 spectra maximally towards red. Hence, the Q_y_ absorption of LH1 in WT Ca^2+^ containing *Tch. tepidum* exhibits at ambient temperature a peak at 914 nm (Fathir et al., 1998), while that in *Trv*. 970 at record long wavelength of 960 nm (Permentier et al., 2001). The depletion of 16 Ca^2+^ ions in the C-terminal domain of LH1 complexes results in significant blue-shifting of their absorbance, see Figure 6A and Table 2. The new structure is less ordered, confirmed by substantial broadening of both the Q_y_ absorption band and IDF. The shift toward higher energies is reversible, so that the original spectra almost precisely recover upon the reconstitution of Ca^2+^ back into the respective protein structures (Imanishi et al., 2019). In contrast, the effect of metal ions (Ca^2+^ or Ba^2+^) on the spectra of LH2 complexes is fairly weak, see Table 3.

We have previously established an enhancement of exciton couplings as the main mechanism of the Ca-facilitated spectral red-shift (Timpmann et al., 2021). These data are reproduced in Figure 6A. Shown in Figure 6B is a fresh result of exchange of Ca^2+^ to Ba^2+^, another group IIA alkaline earth metal with slightly greater ion radius. As seen from spectral responses, the effect of ions exchange is much less dramatic than depletion of the ions. This is reasonable, because the ion replacement is expected only slightly distort the native Ca^2+^ binding sites, while the removal of ions is prone to collapse the sites that may initiate a large-scale restructuring of the protein (Timpmann et al., 2023). The widely separate positions of the blue anisotropy dips observed in case of *Trv.* 970 complexes and the nearly overlapping dips in case of *Tch. tepidum* complexes support these initial conclusions.

**Tertiary-structure hydrogen-bonding effects**. Hydrogen bonding applied at different stages of protein folding is vital for stabilization of the protein. A few well-defined tertiary structure H-bonds have been identified, which are instrumental in color tuning of Q_y_ bands in LH1 and LH2 complexes (Fowler et al., 1994; Sturgis et al., 1997; Sturgis and Robert, 1997; Kangur et al., 2008; Uyeda et al., 2010; Freiberg et al., 2012a). These first and foremost concern the bonds formed between the conserved tryptophan (Trp) and/or tyrosine (Tyr) protein residues and the C3 acetyl carbonyl group of BChls. According to recent theoretical evaluations, the band shifts created are due to joint effect of the dihedral angle of the acetyl moiety and the shuttling of the proton in the H-bond (De Vico et al., 2018).

Any adjustment of the H-bonding pattern necessarily involves transformation of the ground and excited electronic state structures of the excitonically coupled aggregates, hence their exciton properties. A straightforward way to demonstrate this is by direct comparison of the exciton state bandwidths, as revealed by fluorescence excitation anisotropy (data in Tables 2, 3).

Figure 7 provides examples of anisotropy spectra in case of WT and various H-bond mutant LH1-RC and LH2 complexes from *Rba. sphaeroides*. In the LH1-RC complex, one of these mutations (αTrp_+11_Phe, termed α-mutant in Figure 7A), replaces the tryptophan that H-bonds to the C3 acetyl carbonyl group of one of the BChls in a αβ-BChl_2_ apoprotein unit, disrupting the H-bond, while another (βTrp_+9_Phe, β-mutant), disrupts the H-bond to the C3 acetyl carbonyl of the pairing BChl. Double mutant core complexes with replacements in both sites are structurally compromised and are unstable in detergent. For LH2 only membrane-embedded complexes are available carrying the H-bond breaking single (αTyr44Phe, α-mutant) or double (αTyr44Phe-αTyr45Leu, αβ-mutant) residue replacements.

In agreement with the previous knowledge (Fowler et al., 1994), the disruption of particular H-bonds results in blue-shifting of the absorption spectra in comparison with the spectrum of the WT complex. With one notable exception, these spectral shifts are according to expectation accompanied by exciton band narrowing. Except the β-mutant of LH1-RC-pufX, which shows broadening, not narrowing of the exciton bandwidth. Although quantitative evaluation of this effect awaits to be done, the shift of the blue side anisotropy dip allows to conclude that the replacement of the residue at the β_+9_ position causes greater amount of structural rearrangements in LH1 than the replacement at the α_+11_ position, confirming a prior notion obtained from analyses of Raman spectra (Sturgis et al., 1997).

**Carotenoid depletion or exchange**. The significance of carotenoids in the assembly and reinforcement of light-harvesting protein structures is widely recognized (Loach and Parkes-Loach, 2008). Yet the role carotenoids play in modulation of the light-harvesting exciton properties is less well documented. Here, we have been assessing the consequences of carotenoid exchange or depletion in a number of bacterial light-harvesting complexes. The results obtained are collected in Table 2 and Table 3, being also partially illustrated by Supplementary Figure 2.

Supplementary Figure 2 shows that the depletion of carotenoids from either LH1 or LH2 complexes has by far greater impact on light-harvesting excitons than their exchange. The exchange such as demonstrated in Supplementary Figure 2A generally well keeps the structure of the exciton band. However, collapse of the voids subsequent to removal of carotenoid and the accompanying relaxation of the B850/B875 pigment assembles may (as shown in Supplementary Figure 2B) or may not (Supplementary Figure 2C) save the intact exciton structure.

### 3.4 Validation of the top exciton band edge by CD and fluorescence excitation spectroscopy

As it was presented in Introduction, attempts have been made to reveal the electronic structure of BChl excitons in LH2 and LH1 complexes by other experimental techniques, apart from polarized fluorescence spectroscopy. Especially CD method has been popular, because the structure-based model calculations promise clear CD signatures related to both red and blue exciton band edges of light-harvesting complexes that contain cyclic B850/B875 aggregates of BChls. These attempts, however, have all failed in intact peripheral and core light-harvesting units. Not so much because the expected weakness of the CD signal corresponding to the top of the exciton band, but more typically because of unfortunate spectral overlaps with other complements of these units such as RC in LH1-RC or B800 in LH2, which both overwhelmingly contribute into the CD spectra in the spectral range of interest.

Since the efforts to fix the LH2 complexes sufficiently free of B800 pigments proved unsuccessful, we were paying attention to purified LH1 complexes free of RC. In these complexes, a weak negative CD band was previously observed around 754 nm at 77 K and around 763 nm at room temperature (Georgakopoulou et al., 2006b). According to exciton modeling, these features were ascribed to the high-energy exciton component of the Q_y_ band.

**FIGURE 8:**
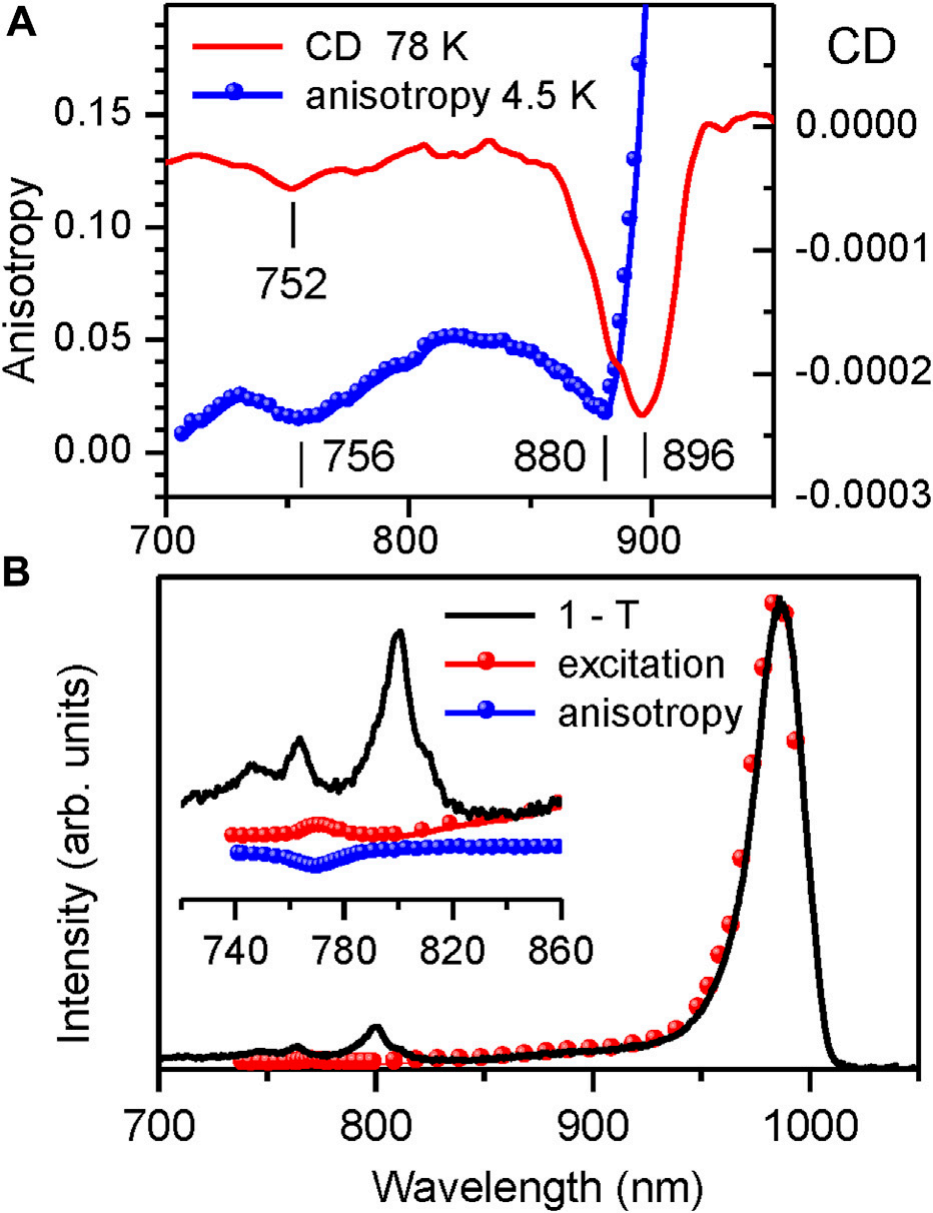
**(A)** Comparison of the CD spectrum (continuous line, recorded at 77 K) and the fluorescence excitation anisotropy spectrum (scattered data points, recorded at 4.5 K (same data as in Figure 4B)) of Q_y_ excitons in detergent-isolated LH1 complexes from *Rba. sphaeroides*. **(B)** Comparison of the absorptance (1–T, black line) and fluorescence excitation (red dots, recorded at 995–1,040 nm) spectra of the LH1-RC complex from Trv. 970 at 4.5 K. The inset shows detailed view of the congested spectral region around the top of the LH1 exciton band with dominating absorption by RC pigments. Shown with scattered blue rings is the fluorescence anisotropy data.

Figure 8A confronts the CD spectrum of the LH1 complex from *Rba. sphaeroides* measured in this work at 77 K with its fluorescence anisotropy spectrum recorded at 4.5 K. Let us note that the CD spectrum of our sample precisely reproduces the already published spectra (Georgakopoulou et al., 2006a; Georgakopoulou et al., 2006b). The close match observed between the location of the CD and anisotropy dips reconfirms that the latter can be considered as an adequate measure of the top exciton band position.

The measurements of fluorescence excitation spectra provide yet another independent validation of the fluorescence anisotropy technique. Demonstrated in Figure 8B is the experimental determination of the top exciton band edge in the intact LH1-RC core complex from *Trv*. 970, in which case the CD method fails totally. The inset represents the blow-up version of the figure in the spectral region of the LH1 exciton band top, where the 1–T spectrum is dominated by the RC absorbance.

As seen, the photons absorbed at cryogenic temperatures by the RC get trapped and do not practically contribute into the LH1 exciton fluorescence. This exposes a rather weak (∼235-fold smaller than the main Q_y_ exciton absorption band at 987 nm) absorption band peaking at 772 nm. Exact overlap with the high-energy anisotropy dip allows this band to be assigned to the exiton states at the very top of the exciton band.

### 3.5 Correlation between the Q_y_ exciton bands and absorption spectra

**FIGURE 9:**
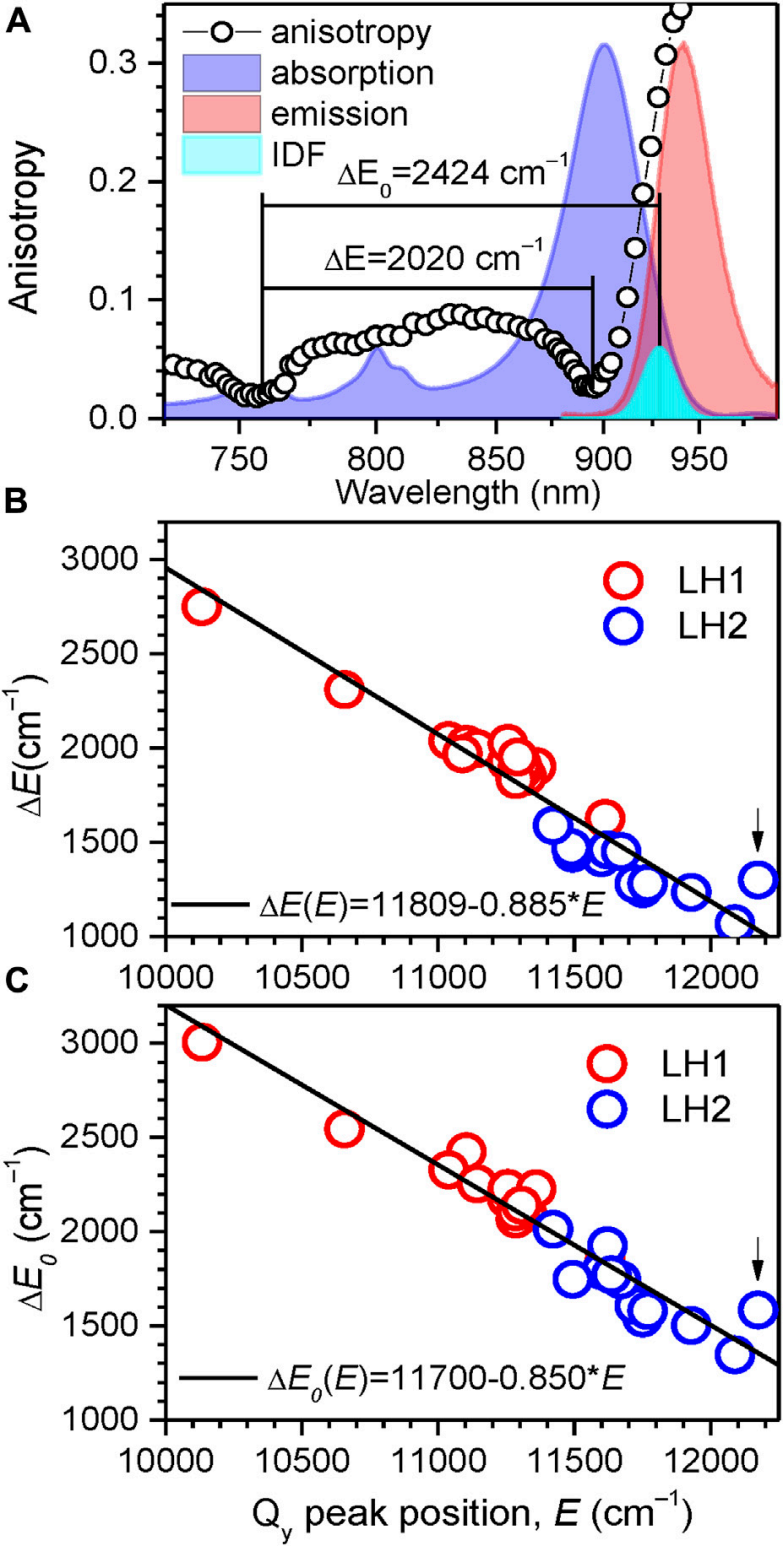
**(A)** The ∆E and ∆E_0_ measures of exciton bandwidth (designated by horizontal lines), illustrated in case of LH1-RC (Ca–) complexes of *Trv*. 970. The approximately Gaussian-shape IDF band (cyan) is arbitrarily scaled with respect to normalized absorption (blue) and fluorescence (red, excited non-resonantly at 407 nm) spectra in the background. **(B, C)** Correlation observed between the exciton bandwidths ∆E **(B)** and ∆E_0_ **(C)**, and the position of the Q_y_ exciton absorption band maximum E_Q_ for the LH1/LH1-RC (red-colored data points) and LH2 (blue-colored data points) complexes. Shown with lines are linear regressions of the data. The data for the LH3 complex from *Rbl. acidophilus* is marked by arrow.

Having determined the boundaries of the Q_y_ exciton state manifolds for all the 33 light-harvesting complexes studied, as represented in Table 2 and Table 3, it would be instrumental to plot the exciton bandwidths as a function of the Q_y_ absorption peak energy, E (Figure 9). The graphs are presented separately for the ∆E (Figure 9B) and ∆E_0_ (Figure 9C) bandwidth definitions. They draw (almost) parallel lines, establishing linear negative correlations between the exciton bandwidths and the E. This means that the complexes with broader bandwidths (with stronger coupled excitons) are adapted to absorb lower-energy (redder) light and *vice versa*. A spectral division of the complexes into two major groups (LH1/LH1-RC core complexes and LH2 peripheral complexes) can also be clearly seen in Figures 9B, C. In energy terms, the color-tuning range of core complexes (1,481 cm^–1^) is almost twice greater than that of LH2 complexes (752 cm^–1^).

The ∆E_0_ exciton bandwidths as a function of E well follow a linear function: ∆E_0_ = 11,700–0.850 × E. A great practical value of this insight is that exciton bandwidths (thus the exciton couplings) and their changes can be readily evaluated with reasonable accuracy using simple measurements of absorption spectra. We have verified that the data that appear deviating from the above linear law correspond to structurally modified complexes, the LH3 complex from *Rbl. acidophilus* being an extreme case. Note, however, that this deceivingly simple relationship determined at 4.5 K is not expected to hold at elevated temperatures, because of thermally-induced variations of site energies, exciton coupling energies as well as the structure of the chromoprotein complexes in certain cases (Rätsep et al., 2018). Additional investigations are, therefore, required to find the bandwidth dependences on temperature (Pajusalu et al., 2011; Freiberg et al., 2013).

The exciton bandwidths recoded in the LH1/LH1-RC core complexes vary between 3,005 and 1,857 cm^–1^, while those in LH2 peripheral complexes, between 2,013 and 1,347 cm^–1^. The exciton couplings energies can be deduced from a “rule of thumb” equation, ∆E_0_ ≈ 2 (V_1_+V_2_), of a model dimerized BChl chain in nearest-neighbor approximation, where V_1_ and V_2_ are the intra- and inter-dimer coupling energies. Assuming V_1_ ≈ V_2_, the low-temperature coupling energies may thus reach about 750 cm^–1^ in LH1/LH1-RC and about 500 cm^–1^ in LH2.

The formation of excitons is usually accompanied by sharpening of monomer spectra, a celebrated example being J-aggregates (Hestand and Spano, 2018). The degree of exciton line narrowing is a function of the ratio Γ_m_/V, where Γ_m_ is the width of the inhomogeneously broadened monomer spectrum and V = (V_1_+V_2_)/2. Therefore, it is expected that the complexes with stronger coupling expose narrower IDF, a results of the so-called exciton motional narrowing effect (Knapp, 1984). This qualitative expectation is indeed roughly followed by our data, see Supplementary Figure 4. It also appears reasonable that the relatively weaker-coupled LH2 complexes are more widely spread with respect to the average value of Γ_IDF_. Yet in several cases such as LH1-RC (Ca−) of *Trv*. 970, LH1-RC (Ba+) of *Tch. tepidum*, LH2 of *E. haloalkaliphila*, and all membrane-embedded LH2 complexes of *Rba. sphaeroides*, anomalously broad IDFs (Γ_IDF_ ≥ 200 cm^–1^) are observed, see Table 2 and Table 3.

In the literature, the width Γ_IDF_ has been widely considered as a proper measure of the exciton disorder (Reddy et al., 1992; Purchase and Völker, 2009). This is not quite correct, because in the chromoprotein systems two qualitatively different types of spectral disorders have been identified, one caused by intra-protein (or internal) and second, due to inter-protein (external) variations (Freiberg et al., 1999), whereas only the former component is subject to motional narrowing. The systems with anomalously broad IDFs listed above thus apparently belong into the realm governed by external disorder.

**FIGURE 10:**
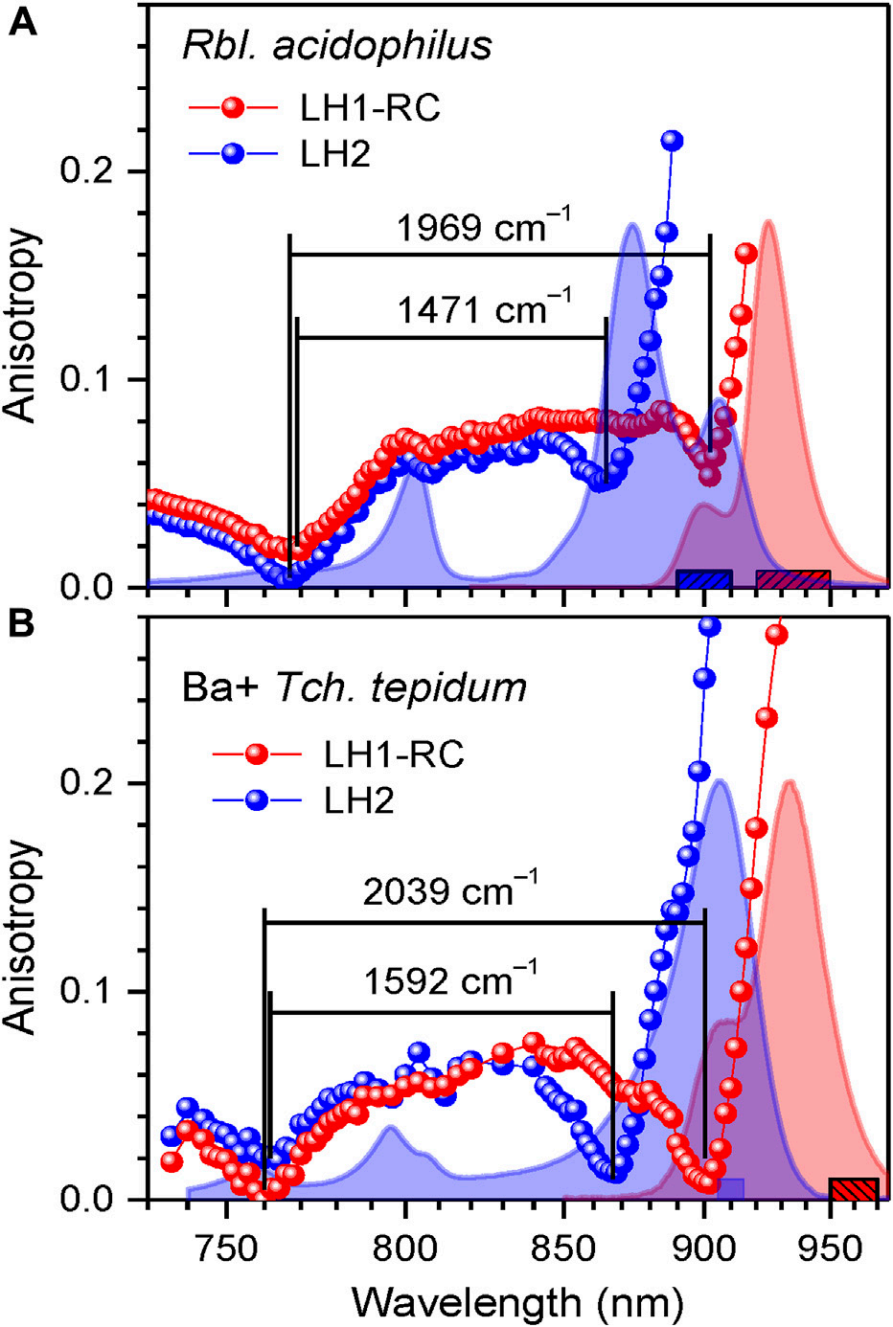
Overlap of exciton bands at 4.5 K of LH1-RC and LH2 complexes in the whole photosynthetic membranes of *Rbl. acidophilus* **(A)** and in a mixture of *Tch. tepidum* complexes containing Ba^2+^ **(B)**. Shown in the background are peak-normalized absorption (blue) and fluorescence (red) spectra of the samples. The colored anisotropy data points are obtained by integrating fluorescence over the corresponding colored bar areas. The lines connecting data points are to lead the eye. See text for further details.

The phototropic bacteria that in addition to core complexes contain peripheral light-harvesting complexes have to carefully tune their respective spectra to achieve optimal transport of excitons towards RCs. As can be seen from Figure 10 and Supplementary Figure 5, the exciton state manifolds in core and peripheral complexes perfectly match each other, whereby the high-energy edges of the exciton state manifolds in LH1 and LH2 nearly coincide. These facts were noticed years ago (Freiberg et al., 2013), but their comprehensiveness wasn’t immediately evident.

## 4 Summary and concluding remarks

Exciton phenomena play a fundamental role in photosynthesis, yet surprisingly, there is a lack of quantitative information obtained directly from experimental studies regarding exciton parameters. The current research addresses this gap by developing an experimental methodology to characterize excitons in cyclic light-harvesting chromoprotein complexes found in photosynthetic purple bacteria. By conducting measurements on 17 core complexes and 16 peripheral complexes, we establish a robust linear correlation between the exciton bandwidth and the positions of the lowest-energy exciton absorption band. This absorption band, associated with Q_y_ electronic transitions in BChl pigments, holds significant importance for photosynthetic exciton transport and trapping. Our findings thus importantly confirm the crucial role of excitons in expanding the spectral range of bacterial light-harvesting. Furthermore, the simple relationship (an “exciton ruler”) identified across the entire class of photosynthetic purple bacterial species enables valuable benchmarking of calculated exciton parameters through straightforward absorption spectrum measurements.

The fluorescence excitation anisotropy method reveals the sensitivity of the blue anisotropy dip to structural changes in the light-harvesting complexes, providing an added advantage. The observation that most modifications to the chromoproteins result in a reduction of exciton bandwidth suggests that the inherent structures of purple bacterial light-harvesting complexes are optimally designed for their function.

## Data Availability

The original contributions presented in the study are included in the article/Supplementary Material, further inquiries can be directed to the corresponding author.

## References

[B1] Alden R. G. Johnson E. Nagarajan V. Parson W. W. Law C. J. Cogdell R. G. (1997). Calculations of spectroscopic properties of the LH2 bacteriochlorophyll - protein antenna complex from *Rhodopseudomonas acidophila* . J. Phys. Chem. B 101, 4667–4680. 10.1021/jp970005r

[B2] Ashikhmin A. Makhneva Z. Moskalenko A. (2014). The LH2 complexes are assembled in the cells of purple sulfur bacterium *Ectothiorhodospira haloalkaliphila* with inhibition of carotenoid biosynthesis. Photosyn. Res. 119, 291–303. 10.1007/s11120-013-9947-6 24163008

[B3] Beekman L. M. P. Frese R. N. Fowler G. J. S. Ortiz de Zarate I. Cogdell R. J. van Stokkum I. (1997). Characterization of the light-harvesting antennas of photosynthetic purple bacteria by Stark spectroscopy. 2. LH2 complexes: influence of the protein environment. J. Phys. Chem. B 101, 7293–7301. 10.1021/jp963447w

[B4] Blankenship R. E. (2002). Molecular mechanisms of photosynthesis. Oxford, United Kingdom: Blackwell Science.

[B5] Bourne-Worster S. Feighan O. Manby F. R. (2023). Charge transfer as a mechanism for chlorophyll fluorescence concentration quenching. Proc. Natl. Acad. Sci. U.S.A. 120, e2210811120. 10.1073/pnas.2210811120 36689657 PMC9945999

[B6] Bowyer J. R. Hunter C. N. Ohnishi T. Niederman R. A. (1985). Photosynthetic membrane development in *Rhodopseudomonas sphaeroides.* Spectral and kinetic characterization of redox components of light-driven electron flow in apparent photosynthetic membrane growth initiation sites. J. Biol. Chem. 260, 3295–3304. 10.1016/S0021-9258(19)83620-7 2982855

[B7] Cao P. Bracun L. Yamagata A. Christianson B. M. Negami T. Zou B. (2022). Structural basis for the assembly and quinone transport mechanisms of the dimeric photosynthetic RC–LH1 supercomplex. Nat. Commun. 13. 10.1038/s41467-022-29563-3 PMC9007983 35418573

[B8] Cignoni E. Slama V. Cupellini L. Mennucci B. (2022). The atomistic modeling of light-harvesting complexes from the physical models to the computational protocol. J. Chem. Phys. 156, 120901. 10.1063/5.0086275 35364859

[B9] Cogdell R. J. Gall A. Köhler J. (2006). The architecture and function of the light-harvesting apparatus of purple bacteria: from single molecules to *in vivo* membranes. Quart. Rev. Biophys. 39, 227–324. 10.1017/S0033583506004434 17038210

[B10] Cogdell R. J. Hawthornthwaite A. M. (1993). “Preparation, purification, and crystallization of purple bacteria antenna complexes,” in Photosynthetic reaction centers. Editors Deisenhofer, J. Norris J. R. (San Diego: Academic Press), 23–42.

[B11] Cogdell R. J. Howard T. D. Isaacs N. W. McLuskey K. Gardiner A. T. (2002). Structural factors which control the position of the Qy absorption band of bacteriochlorophyll a in purple bacterial antenna complexes. Photosyn. Res. 74, 135–141. 10.1023/a:1020995224156 16228551

[B13] Dahlberg P. D. Ting P.-C. Massey S. C. Martin E. C. Hunter C. N. Engel G. S. (2016). Electronic structure and dynamics of higher-lying excited states in light harvesting complex 1 from *Rhodobacter sphaeroides* . *J. Phys. Chem*. A 120, 4124–4130. 10.1021/acs.jpca.6b04146 27232937 PMC5668141

[B14] Davydov A. S. (1971). Theory of molecular excitons. New York: Plenum Press.

[B15] De Vico L. Anda A. Osipov V. A. Madsen A. Ø. Hansen T. (2018). Macrocycle ring deformation as the secondary design principle for light-harvesting complexes. Proc. Natl. Acad. Sci. U.S.A. 115, E9051–E9057. 10.1073/pnas.1719355115 30194231 PMC6166793

[B16] Fassioli F. Dinshaw R. Arpin P. C. Scholes G. D. (2014). Photosynthetic light harvesting: excitons and coherence. J. R. Soc. Interface 11, 20130901. 10.1098/rsif.2013.0901 24352671 PMC3899860

[B17] Fathir I. Ashikaga M. Tanaka K. Katano T. Nirasawa T. Kobayashi M. (1998). Biochemical and spectral characterization of the core light harvesting complex 1 (LH1) from the thermophilic purple sulfur bacterium *Chromatium tepidum* . Photosyn. Res. 58, 192–202. 10.1023/A:1006146713751

[B18] Fowler G. J. S. Sockalingum G. D. Robert B. Hunter C. N. (1994). Blue shifts in bacteriochlorophyll absorbance correlate with changed hydrogen bonding patterns in light-harvesting 2 mutants of *Rhodobacter sphaeroides* with alterations at alpha-Tyr-44 and alpha-Tyr-45. Biochem. J. 299, 695–700. 10.1042/bj2990695 8192657 PMC1138076

[B19] Freiberg A. Chenchiliyan M. Rätsep M. Timpmann K. (2016). Spectral and kinetic effects accompanying the assembly of core complexes of Rhodobacter sphaeroides. Biochim. Biophys. Acta - Bioenerg. 1857, 1727–1733. 10.1016/j.bbabio.2016.08.001 27514285

[B20] Freiberg A. Godik V. I. Pullerits T. Timpman K. (1989). Picosecond dynamics of directed excitation transfer in spectrally heterogeneous light-harvesting antenna of purple bacteria. Biochim. Biophys. Acta – Bioenerg. 973, 93–104. 10.1016/S0005-2728(89)80407-4

[B21] Freiberg A. Kangur L. Olsen J. Hunter C. N. (2012). Structural implications of hydrogen-bond energetics in membrane proteins revealed by high-pressure spectroscopy. Biophys. J. 103, 2352–2360. 10.1016/j.bpj.2012.10.030 23283234 PMC3514530

[B22] Freiberg A. Pajusalu M. Rätsep M. (2013). Excitons in intact cells of photosynthetic bacteria. J. Phys. Chem. B 117, 11007–11014. 10.1021/jp3098523 23379598

[B23] Freiberg A. Rätsep M. Timpmann K. (2012b). A comparative spectroscopic and kinetic study of photoexcitations in detergent-isolated and membrane-embedded LH2 light-harvesting complexes. Biochem. Biophys. Acta – Bioenerg. 1817, 1471–1482. 10.1016/j.bbabio.2011.11.019 22172735

[B24] Freiberg A. Rätsep M. Timpmann K. Trinkunas G. Woodbury N. W. (2003). Self-trapped excitons in LH2 antenna complexes between 5 K and ambient temperature. J. Phys. Chem. B 107, 11510–11519. 10.1021/jp0344848

[B25] Freiberg A. Timpmann K. Ruus R. Woodbury N. W. (1999). Disordered exciton analysis of linear and nonlinear absorption spectra of antenna bacteriochlorophyll aggregates: LH2-only mutant chromatophores of *Rhodobacter sphaeroides* at 8 K under spectrally selective excitation. J. Phys. Chem. B 103, 10032–10041. 10.1021/jp991676n

[B26] Freiberg A. Timpmann K. Trinkunas G. (2010). Spectral fine-tuning in excitonically coupled cyclic photosynthetic antennas. Chem. Phys. Lett. 500, 111–115. 10.1016/j.cplett.2010.09.084

[B27] Fresch E. Meneghin E. Agostini A. Paulsen H. Carbonera D. Collini E. (2020). How the protein environment can tune the energy, the coupling, and the ultrafast dynamics of interacting chlorophylls: the example of the water-soluble chlorophyll protein. J. Phys. Chem. Lett. 11, 1059–1067. 10.1021/acs.jpclett.9b03628 31952446 PMC7995254

[B28] Gall A. Sogaila E. Gulbinas V. Ilioaia O. Robert B. Valkunas L. (2010). Spectral dependence of energy transfer in wild-type peripheral light-harvesting complexes of photosynthetic bacteria. Biochem. Biophys. Acta – Bioenerg. 1797, 1465–1469. 10.1016/j.bbabio.2010.05.004 20470750

[B29] Gardiner A. T. Naydenova K. Castro-Hartmann P. Nguyen-Phan T. C. Russo C. J. Sader K. (2021). The 2.4 Å cryo-EM structure of a heptameric light-harvesting 2 complex reveals two carotenoid energy transfer pathways. Sci. Adv. 7, eabe4650. 10.1126/sciadv.abe4650 33579696 PMC7880592

[B30] Gardiner A. T. Nguyen-Phan T. C. Cogdell R. J. (2020). A comparative look at structural variation among RC–LH1 ‘Core’ complexes present in anoxygenic phototrophic bacteria. Photosyn. Res. 145, 83–96. 10.1007/s11120-020-00758-3 PMC7423801 32430765

[B31] Georgakopoulou S. van der Zwan G. Olsen J. D. Hunter C. N. Niederman R. A. van Grondelle R. (2006a). Investigation of the effects of different carotenoids on the absorption and CD signals of light harvesting 1 complexes. J. Phys. Chem. B 110, 3354–3361. 10.1021/jp0517955 16494350

[B32] Georgakopoulou S. van Grondelle R. van der Zwan G. (2006b). Explaining the visible and near-infrared circular dichroism spectra of light-harvesting 1 complexes from purple bacteria: a modeling study. J. Phys. Chem. B 110, 3344–3353. 10.1021/jp051794c 16494349

[B33] Grimm B. Porra R. J. Rüdiger W. Scheer H. (Editors) (2006). Chlorophylls and bacteriochlorophylls. Biochemistry, biophysics, functions and applications (Dordrecht: Springer). 10.1007/1-4020-4516-6

[B34] Hestand N. J. Spano F. C. (2018). Expanded theory of H- and J-molecular aggregates: the effects of vibronic coupling and intermolecular charge transfer. Chem. Rev. 118, 7069–7163. 10.1021/acs.chemrev.7b00581 29664617

[B35] Hu X. Ritz T. Damjanovic A. Autenrieth F. Schulten K. (2002). Photosynthetic apparatus of purple bacteria. Quart. Rev. Biophys. 35, 1–62. 10.1017/s0033583501003754 11997980

[B36] Hunter C. N. Daldal F. Thurnauer M. C. Beatty J. T. (Editors) (2008). “The purple phototrophic bacteria,” Advances in photosynthesis and respiration (Dordrecht: Springer).

[B37] Hunter C. N. Kramer H. J. M. van Grondelle R. (1985). Linear dichroism and fluorescence emission of antenna complexes during photosynthetic unit assembly in *Rhodopseudomonas sphaeroides. Biochim. Biophys* . Acta – Bioenerg. 807, 44–51. 10.1016/0005-2728(85)90051-9

[B38] Imanishi M. Takenouchi M. Takaichi S. Nakagawa S. Saga Y. Takenaka S. (2019). A dual role for Ca^2+^ in expanding the spectral diversity and stability of light-harvesting 1 reaction center photocomplexes of purple phototrophic bacteria. Biochem. 58, 2844–2852. 10.1021/acs.biochem.9b00351 31145583

[B39] Jang S. J. Mennucci B. (2018). Delocalized excitons in natural light-harvesting complexes. Rev. Mod. Phys. 90, 035003. 10.1103/RevModPhys.90.035003

[B40] Jones M. R. Fowler G. J. S. Gibson L. C. D. Grief G. G. Olsen J. D. Crielaard W. (1992). Mutants of *Rhodobacter sphaeroides* lacking one or more pigment-protein complexes and complementation with reaction-centre, LH1, and LH2 genes. Mol. Microbiol. 6, 1173–1184. 10.1111/j.1365-2958.1992.tb01556.x 1588816

[B41] Kangur L. Timpmann K. Freiberg A. (2008). Stability of integral membrane proteins against high hydrostatic pressure: the LH2 and LH3 antenna pigment-protein complexes from photosynthetic bacteria. J. Phys. Chem. B 112, 7948–7955. 10.1021/jp801943w 18537288

[B42] Kania A. Fiedor L. (2006). Steric control of bacteriochlorophyll ligation. J. Am. Chem. Soc. 128, 454–458. 10.1021/ja055537x 16402831

[B43] Kereïche S. Bourinet L. Keegstra W. Arteni A. A. Verbavatz J.-M. Boekema E. J. (2008). The peripheral light-harvesting complexes from purple sulfur bacteria have different ‘ring’ sizes. FEBS Lett. 582, 3650–3656. 10.1016/j.febslet.2008.09.050 18840433

[B44] Kimura Y. Tani K. Madigan M. T. Wang-Otomo Z.-Y. (2023). Advances in the spectroscopic and structural characterization of core light-harvesting complexes from purple phototrophic bacteria. J. Phys. Chem. B 127, 6–7. 10.1021/acs.jpcb.2c06638 36594654

[B45] Knapp E. W. (1984). Lineshapes of molecular aggregates. Exchange narrowing and intersite correlation. Chem. Phys. 85, 73–82. 10.1016/S0301-0104(84)85174-5

[B46] Koolhaas M. H. C. Frese R. N. Fowler G. J. S. Bibby T. A. Georgakopoulou S. van der Zwan G. (1998). Identification of the upper exciton component of the B850 bacteriochlorophylls of the LH2 antenna complex, using a B800-free mutant of *Rhodobacter sphaeroides* . Biochem. 37, 4693–4698. 10.1021/bi973036l 9548732

[B47] Kunz R. Timpmann K. Southall J. Cogdell R. J. Köhler J. Freiberg A. (2013). Fluorescence-excitation and emission spectra from LH2 antenna complexes of *Rhodopseudomonas acidophila* as a function of the sample preparation conditions. J. Phys. Chem. B 117, 12020–12029. 10.1021/jp4073697 24033126

[B48] Leiger K. Linnanto J. M. Freiberg A. (2020). Establishment of the Qy absorption spectrum of chlorophyll a extending to near-infrared. Molecules 25, 3796. 10.3390/molecules25173796 32825445 PMC7503670

[B49] Leupold D. Stiel H. Ehlert J. Nowak F. Teuchner K. Voigt B. (1999). Photophysical characterization of the B800-depleted light harvesting complex B850 of *Rhodobacter sphaeroides* - implications to the ultrafast energy transfer 800 -> 850 nm. Chem. Phys. Lett. 301, 537–545. 10.1016/S0009-2614(99)00061-5

[B50] Limantara L. Sakamoto S. Koyama Y. Nagea H. (1997). Effects of nonpolar and polar solvents on the Qx and Qy energies of bacteriochlorophyll a and bacteriopheophytin a. Photochem. Photobiol. 65, 330–337. 10.1111/j.1751-1097.1997.tb08566.x

[B51] Loach P. A. Parkes-Loach P. S. (2008). “Structure-function relationships in bacterial light-harvesting complexes investigated by reconstitution techniques,” in The purple photosynthetic bacteria. Editors Hunter C. N. Daldal F. Thurnauer M. C. Beatty J. T. (Dordrecht: Springer), 181–198.

[B52] May V. Kühn O. (2008). Charge and energy transfer dynamics in molecular systems. Weinheim: Wiley.

[B53] Mirkovic T. Ostroumov E. E. Anna J. M. van Grondelle R. Govindjee Scholes G. D. (2017). Light absorption and energy transfer in the antenna complexes of photosynthetic organisms. Chem. Rev. 117, 249–293. 10.1021/acs.chemrev.6b00002 27428615

[B54] Moskalenko A. Toropygina O. Kuznetsova N. (1996). Does the B820 subcomplex of the B880 complex retain carotenoids? Z. Naturforsch. C 51, 309–318. 10.1515/znc-1996-5-608

[B55] Nottoli M. Jurinovich S. Cupellini L. Gardiner A. T. Cogdell R. Mennucci B. (2018). The role of charge-transfer states in the spectral tuning of antenna complexes of purple bacteria. Photosyn. Res. 137, 215–226. 10.1007/s11120-018-0492-1 29502240

[B56] Novoderezhkin V. I. Razjivin A. P. (1995). Theoretical study of circular dichroism of the light-harvesting antenna of photosynthetic purple bacteria: a consideration of exciton interactions and energy disorder. Photochem. Photobiol. 62, 1035–1040. 10.1111/j.1751-1097.1995.tb02405.x

[B57] Olsen J. D. Sockalingum G. D. Robert B. Hunter C. N. (1994). Modification of a hydrogen bond to a bacteriochlorophyll a molecule in the light-harvesting 1 antenna of *Rhodobacter sphaeroides* . Proc. Natl. Acad. Sci. U.S.A. 91, 7124–7128. 10.1073/pnas.91.15.7124 8041757 PMC44351

[B58] Pajusalu M. Rätsep M. Trinkunas G. Freiberg A. (2011). Davydov splitting of excitons in cyclic bacteriochlorophyll a nanoaggregates of bacterial light-harvesting complexes between 4.5 and 263 K. ChemPhysChem 12, 634–644. 10.1002/cphc.201000913 21275034

[B59] Permentier H. P. Neerken S. Overmann J. Amesz J. (2001). A bacteriochlorophyll a antenna complex from purple bacteria absorbing at 963 nm. Biochem. 40, 5573–5578. 10.1021/bi0024308 11331023

[B60] Pflock T. Dezi M. Venturoli G. Cogdell R. J. Köhler J. Ollerich S. (2008). Comparison of the fluorescence kinetics of detergent-solubilised and membrane-reconstituted LH2 complexes from. Rps. acidophila Rb. sphaeroides. Photosyn. Res. 95, 291–298. 10.1007/s11120-007-9245-2 17912609

[B61] Pugh R. J. McGlynn P. Jones M. R. Hunter C. N. (1998). The LH1-RC core complex of *Rhodobacter sphaeroides:* interaction between components, time-dependent assembly, and topology of the PufX protein. Biochim. Biophys. Acta – Bioenerg. 1366, 301–316. 10.1016/S0005-2728(98)00131-5 9814844

[B62] Purchase R. Völker S. (2009). Spectral hole burning: examples from photosynthesis. Photosyn. Res. 101, 245–266. 10.1007/s11120-009-9484-5 PMC2744831 19714478

[B63] Qian P. Croll T. I. Hitchcock A. Jackson P. J. Salisbury J. H. Castro-Hartmann P. (2021a). Cryo-EM structure of the dimeric *Rhodobacter sphaeroides* RC-LH1 core complex at 2.9 Å: the structural basis for dimerisation. Biochem. J. 478, 3923. 10.1042/bcj20210696 34622934 PMC8652583

[B64] Qian P. Swainsbury D. J. K. Croll T. I. Salisbury J. H. Martin E. C. Jackson P. J. (2021b). Cryo-EM structure of the monomeric *Rhodobacter sphaeroides* RC–LH1 core complex at 2.5 Å. Biochem. J. 478, 3775–3790. 10.1042/bcj20210631 34590677 PMC8589327

[B65] Rätsep M. Muru R. Freiberg A. (2018). High temperature limit of photosynthetic excitons. Nat. Commun. 9, 99. 10.1038/s41467-017-02544-7 29311621 PMC5758513

[B66] Rätsep M. Timpmann K. Kawakami T. Wang-Otomo Z.-Y. Freiberg A. (2017). Spectrally selective spectroscopy of native Ca-containing and Ba-substituted LH1-RC core complexes from *Thermochromatium tepidum* . J. Phys. Chem. B 121, 10318–10326. 10.1021/acs.jpcb.7b07841 29058423

[B67] Razjivin A. Solov’ev A. Kompanets V. Chekalin S. Moskalenko A. Lokstein H. (2019). The origin of the “dark” absorption band near 675 nm in the purple bacterial core light-harvesting complex LH1: two-photon measurements of LH1 and its subunit B820. Photosyn. Res. 140, 207–213. 10.1007/s11120-018-0602-0 30411209

[B68] Reddy N. R. S. Picorel R. Small G. J. (1992). B896 and B870 components of the *Rhodobacter sphaeroides* antenna: a hole burning study. J. Phys. Chem. 96, 6458–6464. 10.1021/j100194a065

[B69] Reddy N. R. S. Small G. J. (1991). Hole burning as a probe of exciton bandwidths in amorphous solids. J. Chem. Phys. 94, 7545–7546. 10.1063/1.460186

[B70] Reimers J. R. Rätsep M. Freiberg A. (2020). Asymmetry in the Q_y_ fluorescence and absorption spectra of chlorophyll *a* pertaining to exciton dynamics. Front. Chem. 8, 588289. 10.3389/fchem.2020.588289 33344415 PMC7738624

[B71] Sauer K. Austin L. A. (1978). Bacteriochlorophyll-protein complexes from the light-harvesting antenna of photosynthetic bacteria. Biochem. 17, 2011–2019. 10.1021/bi00603a033 418797

[B72] Scherz A. Parson W. W. (1984). Oligomers of bacteriochlorophyll and bacteriopheophytin with spectroscopic properties resembling those found in photosynthetic bacteria. Biochim. Biophys. Acta – Bioenerg. 766, 653–665. 10.1016/0005-2728(84)90127-0

[B73] Scholes G. D. Fleming G. R. Olaya-Castro A. van Grondelle R. (2011). Lessons from nature about solar light harvesting. Nat. Chem. 3, 763–774. 10.1038/nchem.1145 21941248

[B74] Sohail S. H. Dahlberg P. D. Allodi M. A. Massey S. C. Ting P.-C. Martin E. C. (2017). Broad manifold of excitonic states in light-harvesting complex 1 promotes efficient unidirectional energy transfer *in vivo* . J. Chem. Phys. 147, 131101. 10.1063/1.4999057 28987085 PMC5848712

[B75] Stepanenko I. Kompanetz V. Makhneva Z. Chekalin S. Moskalenko A. Razjivin A. (2012). Transient absorption study of two-photon excitation mechanism in the LH2 complex from purple bacterium *Rhodobacter sphaeroides* . J. Phys. Chem. B 116, 2886–2890. 10.1021/jp2033214 22268655

[B76] Sturgis J. N. Olsen J. D. Robert B. Hunter C. N. (1997). Functions of conserved tryptophan residues of the core light-harvesting complex of *Rhodobacter sphaeroides* . Biochem. 36, 2772–2778. 10.1021/bi962524a 9062104

[B77] Sturgis J. N. Robert B. (1997). Pigment binding-site and electronic properties in light-harvesting proteins of purple bacteria. J. Phys. Chem. B 101, 7227–7231. 10.1021/jp963363n

[B78] Suzuki H. Hirano Y. Kimura Y. Takaichi S. Kobayashi M. Miki K. (2007). Purification, characterization and crystallization of the core complex from thermophilic purple sulfur bacterium *Thermochromatium tepidum. Biochim. Biophys* . Acta – Bioenerg. 1767, 1057–1063. 10.1016/j.bbabio.2007.06.002 17658456

[B79] Swainsbury D. J. K. Faries K. M. Niedzwiedzki D. M. Martin E. C. Flinders A. J. Canniffe D. P. (2019). Engineering of B800 bacteriochlorophyll binding site specificity in the *Rhodobacter sphaeroides* LH2 antenna. Biochim. Biophys. Acta – Bioenerg. 1860, 209–223. 10.1016/j.bbabio.2018.11.008 30414933 PMC6358721

[B92] Tani K. Kanno R. Makino Y. Hall M. Takenouchi M. Imanishi M. (2020). Cryo-EM structure of a Ca^2+^-bound photosynthetic LH1-RC complex containing multiple αβ-polypeptides. Nat. Commun. 11, 4955. 10.1038/s41467-020-18748-3 33009385 PMC7532537

[B80] Taniguchi M. Lindsey J. S. (2021). Absorption and fluorescence spectral database of chlorophylls and analogues. Photochem. Photobiol. 97, 136–165. 10.1111/php.13319 32757305

[B81] Timpmann K. Kangur L. Freiberg A. (2023). Hysteretic pressure dependence of Ca^2+^ binding in LH1 bacterial membrane chromoproteins. J. Phys. Chem. B 127, 456–464. 10.1021/acs.jpcb.2c05938 36608327

[B82] Timpmann K. Katiliene Z. Woodbury N. W. Freiberg A. (2001). Exciton self-trapping in one-dimensional photosynthetic antennas. J. Phys. Chem. B 105, 12223–12225. 10.1021/jp011147v

[B83] Timpmann K. Rätsep M. Kangur L. Lehtmets A. Wang-Otomo Z.-Y. Freiberg A. (2021). Exciton origin of color-tuning in Ca^2+^-binding photosynthetic bacteria. Int. J. Mol. Sci. 22, 7338. 10.3390/ijms22147338 34298960 PMC8303132

[B84] Timpmann K. Trinkunas G. Olsen J. D. Hunter C. N. Freiberg A. (2004). Bandwidth of excitons in LH2 bacterial antenna chromoproteins. Chem. Phys. Lett. 398, 384–388. 10.1016/j.cplett.2004.09.090

[B85] Timpmann K. Trinkunas G. Qian P. Hunter C. N. Freiberg A. (2005). Excitons in core LH1 antenna complexes of photosynthetic bacteria: evidence for strong resonant coupling and off-diagonal disorder. Chem. Phys. Lett. 414, 359–363. 10.1016/j.cplett.2005.08.094

[B86] Trinkunas G. Freiberg A. (2006). A disordered polaron model for polarized fluorescence excitation spectra of LH1 and LH2 bacteriochlorophyll antenna aggregates. J. Lumin. 119–120, 105–110. 10.1016/j.jlumin.2005.12.060

[B87] Trinkunas G. Zerlauskiene O. Urbonienė V. Chmeliov J. Gall A. Robert B. (2012). Exciton band structure in bacterial peripheral light-harvesting complexes. J. Phys. Chem. B 116, 5192–5198. 10.1021/jp302042w 22480241

[B88] Urboniene V. Vrublevskaja O. Trinkunas G. Gall A. Robert B. Valkunas L. (2007). Solvation effect of bacteriochlorophyll excitons in light-harvesting complex LH2. Biophys. J. 93, 2188–2198. 10.1529/biophysj.106.103093 17513366 PMC1959563

[B89] Uyeda G. Williams J. C. Roman M. Mattioli T. A. Allen J. P. (2010). The influence of hydrogen bonds on the electronic structure of light-harvesting complexes from photosynthetic bacteria. Biochem. 49, 1146–1159. 10.1021/bi901247h 20067231

[B90] van Amerongen H. Valkunas L. van Grondelle R. (2000). Photosynthetic excitons. Singapore: World Scientific. 10.1142/9789812813664

[B91] Zucchelli G. Brogioli D. Casazza A. P. Garlaschi F. M. Jennings R. C. (2007). Chlorophyll ring deformation modulates Qy electronic energy in chlorophyll-protein complexes and generates spectral forms. Biophys. J. 93, 2240–2254. 10.1529/biophysj.107.104554 17513370 PMC1959541

