# Supplementary material for: Dominant role of excitons in photosynthetic color-tuning and light-harvesting
Source: Front Chem. 2023 Oct 16;11:1231431. doi: 10.3389/fchem.2023.1231431 (PMC10613661; doi:10.3389/fchem.2023.1231431)
Supplement: Supplementary file 1 [file DataSheet1.PDF]

# *Supplementary Material*

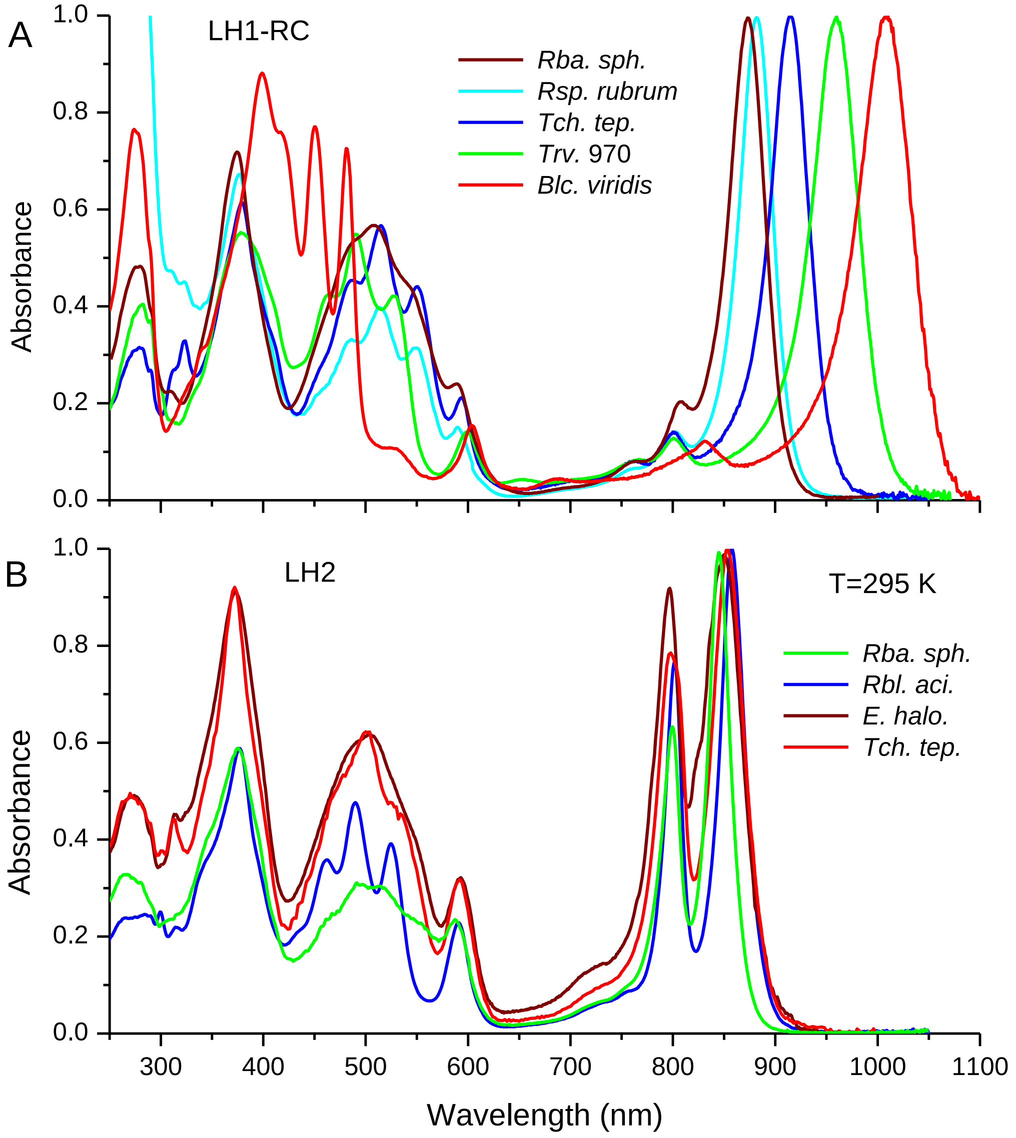


**Supplementary Figure 1.** Broadband absorption spectra of core LH1-RC (A) and peripheral LH2 (B) light-harvesting complexes from wild type purple bacteria recorded at ambient temperature of 295 K.

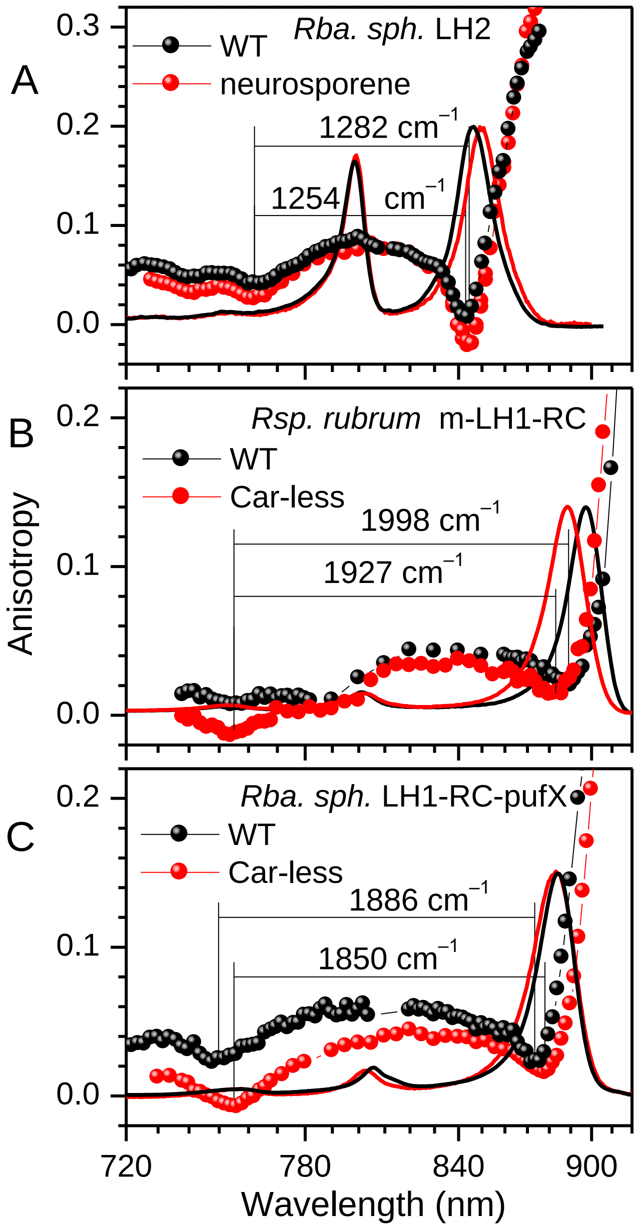


**Supplementary Figure 2.** Comparison of the fluorescence excitation anisotropy spectrum (scatted data points) of the $Q_y$ excitons (A) in wild type LH2 complexes from *Rba. sphaeroides* containing spheroidenone (black) as the main carotenoid with that of complexes containing neurosporene (red), (B) in wild type (black) and carotenoid-less (red) LH1-RC complexes in chromatophores of *Rsp. rubrum*, and (C) in the wild type (black) and carotenoid-less (red) LH1-RC complexes of ΔCrtB mutant chromatophores of *Rba. sphaeroides*. Shown in the background are correspondingly colored absorption spectra of the samples.

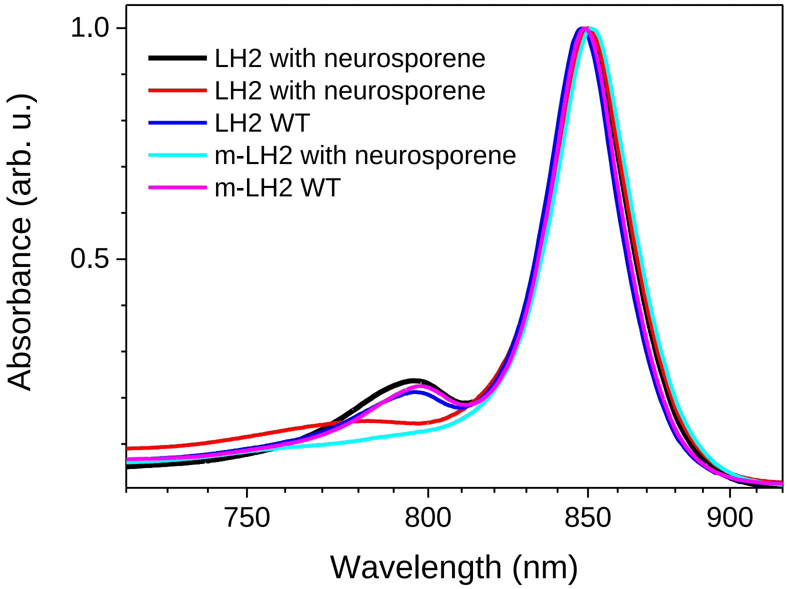


**Supplementary Figure 3.** Peak-normalized absorption spectra of five individual preparations of purified and membrane B850 only LH2 complexes from *Rba. sphaeroides* recorded at ambient temperature. In three of them the WT content of carotenoids was exchanged to neurosporene. Notice the significant deviations of the spectra around 770–810 nm.

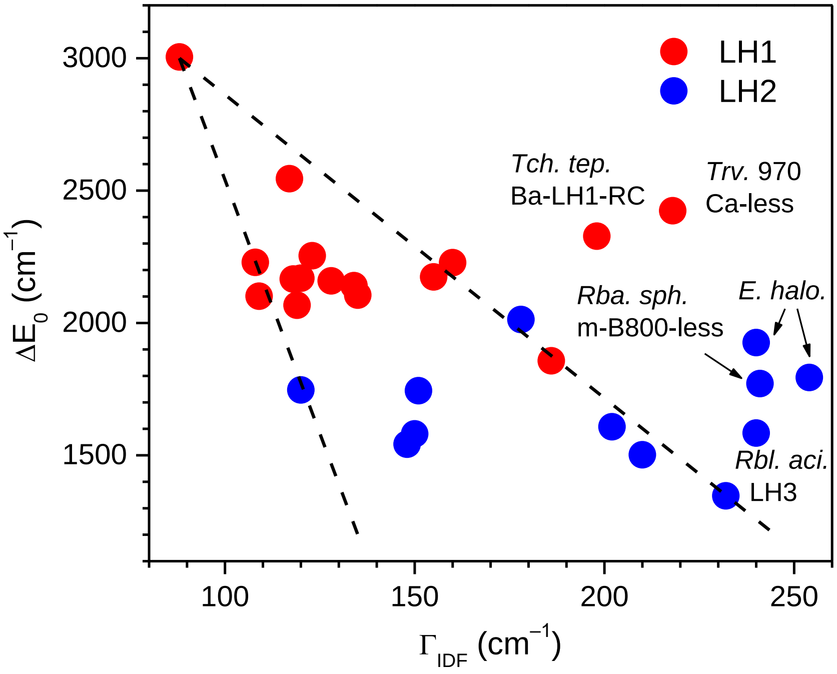


**Supplementary Figure 4.** Correlation between the $\Delta E_0$ exciton bandwidth and $\Gamma_{IDF}$, the width of inhomogeneous distribution function. As seen, most data are concentrated into the area between dashed lines.

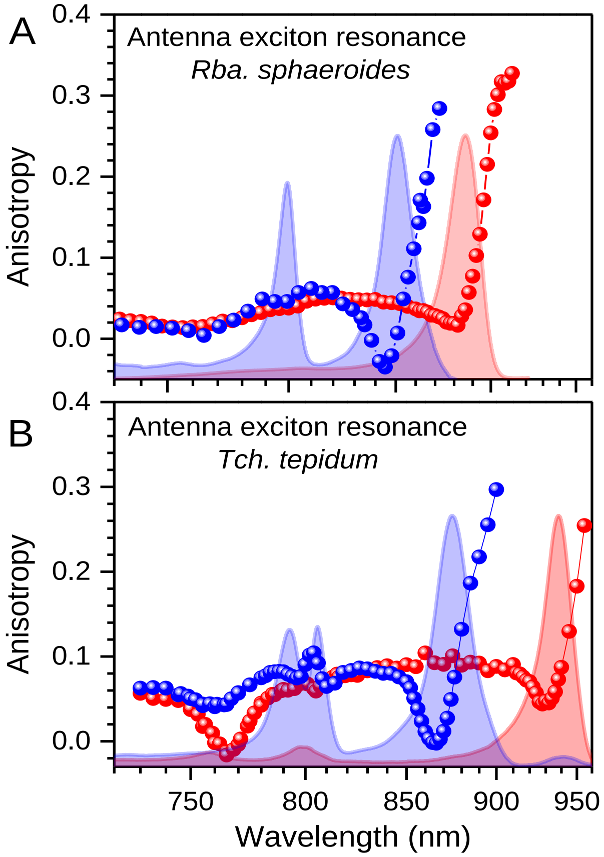


**Supplementary Figure 5.** Matching fluorescence excitation anisotropy spectra of purified LH1/LH1-RC and LH2 complexes from *Rba. sphaeroides* (A) and *Tch. tepidum* (B). Shown also are the peak-normalized absorption spectra of the related complexes.
